# Clustering Deviation Index (CDI): a robust and accurate internal measure for evaluating scRNA-seq data clustering

**DOI:** 10.1186/s13059-022-02825-5

**Published:** 2022-12-27

**Authors:** Jiyuan Fang, Cliburn Chan, Kouros Owzar, Liuyang Wang, Diyuan Qin, Qi-Jing Li, Jichun Xie

**Affiliations:** 1 Department of Biostatistics and Bioinformatics, Duke University School of Medicine, Durham, USA; 2 Center for Human Systems Immunology, School of Medicine, Duke University, Durham, USA; 3 Department of Molecular Genetics and Microbiology, School of Medicine, Duke University, Durham, USA; 4 Department of Immunology, School of Medicine, Duke University, Durham, USA; 5 Clinical Trial Center, National Medical Products Administration Key Laboratory for Clinical Research and Evaluation of Innovative Drugs, West China Hospital, Sichuan University, Chengdu, China; 6 Department of Mathematics, Duke University, Durham, USA

**Keywords:** Single-cell, RNA-sequencing, Clustering, Assessment

## Abstract

Most single-cell RNA sequencing (scRNA-seq) analyses begin with cell clustering; thus, the clustering accuracy considerably impacts the validity of downstream analyses. In contrast with the abundance of clustering methods, the tools to assess the clustering accuracy are limited. We propose a new Clustering Deviation Index (CDI) that measures the deviation of any clustering label set from the observed single-cell data. We conduct in silico and experimental scRNA-seq studies to show that CDI can select the optimal clustering label set. As a result, CDI also informs the optimal tuning parameters for any given clustering method and the correct number of cluster components.

**Supplementary Information:**

The online version contains supplementary material available at 10.1186/s13059-022-02825-5.

## Background

Single-cell RNA-sequencing (scRNA-seq) quantifies the transcriptome of individual cells, allowing us to explore the biological heterogeneity among cells [1]. Thus, scRNA-seq analysis usually begins with cell type clustering. Over the past 5 years, many methods have been developed or re-purposed for scRNA-seq clusterings, such as K-means [2, 3], hierarchical clustering [3], RaceID [4], CIDR [5], SIMLR [6], SCANPY (Louvain algorithm) [7], and Seurat (Louvain algorithm) [8].

The outputs of these clustering methods are cell label sets that assign each cell to a cluster. Different methods usually yield different label sets. Even if we use a given clustering method, we still obtain different label sets by setting different tuning parameters. These different label sets introduce the challenge of choosing the “optimal” label set. One approach to address the challenge is applying a consensus method to these label sets to derive an ensemble label set. However, the ensemble label set is not guaranteed to reflect the underlying cell type structure better than any input label set. Furthermore, different consensus methods often generate different ensemble label sets; thus, the challenge of choosing the optimal label set remains. Therefore, we need a robust and accurate index to score each label set’s deviation (or “goodness-of-fit”); based on the scores, we can select the optimal label set [9, 10].

In general, the evaluating indices can be divided into two categories. The first category consists of external indices, which measure the agreement between candidate and benchmark label sets. The second category consists of internal indices, whose calculation does not depend on a benchmark label set.

External indices include, but are not limited to, the Adjusted Rand Index (ARI) [11], the Fowlkes-Mallows (FM) index [12], and the Normalized Mutual Information (NMI) [13]. External indices utilize the benchmark label set to evaluate the quality of the candidate label set. If a high-quality benchmark label set is available, external indices are more accurate than internal indices, which do not utilize the high-quality benchmark label set. However, external indices cannot be applied when the benchmark label set is unavailable. Moreover, external indices may perform unsatisfyingly when the benchmark label set is of low quality. These practical issues could occur during scRNA-seq clustering.

Internal indices usually use topological or statistical properties to evaluate the quality of a label set. Examples of internal indices include, but are not limited to, Calinski-Harabasz index (CH) [14], Connectivity [15], Davies-Bouldin index (DB) [16], Dunn index [17], Baker-Hubert Gamma index [18], SD index (SD) [19], and Xie-Beni index (XB) [20]. These indices can measure the goodness-of-fit of the candidate label sets for a scRNA-seq dataset. However, when we applied these indices to scRNA-seq clustering, different indices selected different label sets as optimal. Moreover, these internal indices require a long computation time; thus, they are not computationally feasible for large-scale scRNA-seq data.

In addition to internal and external indices, some stability indices evaluate clustering methods based on the clustering stability when the original data are perturbed (resampled, split, subsampled). See [21] for a recent review of these indices. The key idea is to produce perturbed datasets whose distribution is close to the original and apply the clustering methods. The clusters from the original and perturbed data will be similar if the clustering is stable. We do not consider stability indices in our paper for three reasons. First, we aim to find the optimal candidate label set that fits the scRNA-seq dataset. The clustering stability does not necessarily reflect its fitting quality. Second, scRNA-seq data are high-dimensional and sparse. It is challenging to create a perturbed dataset with similar distributions. Especially when the data contain rare cell types, resampling, splitting, or subsampling could change the proportion of rare cell types in the data or even miss rare cell types. Third, the stability indices are usually more computationally intensive to calculate. For large-scale scRNA-seq datasets, applying all clustering methods with a wide range of tuning parameters for the original dataset is already computationally challenging; applying them under various perturbations is impractical.

In this study, we developed a new clustering evaluation index, Clustering Deviation Index (CDI), to quantify the deviation of distributions based on the given label set from the observed single-cell data. CDI is an internal index whose calculation does not rely on the knowledge of the benchmark label set. While other internal indices are designed to evaluate clustering under general situations, CDI is customized for scRNA-seq clustering: it utilizes the UMI distribution properties of scRNA-seq data to calculate the index. As a result, compared with other internal indices, CDI has superior performance in evaluating scRNA-seq clustering and achieves the highest level of agreement with external indices (ARI, FM, and NMI) using the high-quality benchmark label sets. It is also more efficient than other internal indices and works well on large-scale datasets. We applied CDI to multiple simulated and experimental scRNA-seq datasets and successfully selected biologically meaningful clustering labels for all datasets.

## Results

### UMI count distributions

We started by checking the distributions of UMI counts generated by droplet-based protocols for both monoclonal and polyclonal cell populations (Table 1). In many scRNA-seq protocols, UMI is barcoded for each transcript before amplification, leading to more accurate quantification of the transcript counts [22, 23].

**Table 1:** Dataset summary. See 5: 5.4 for the details of these datasets. For each simulated dataset, the actual cell type label set is known. For each experimental dataset, the benchmark label set was obtained by the experimental and bioinformatics analysis process. This process includes fluorescence-activated cell sorting (FACS), known feature gene checking, cell screening, and clustering. Thus, although these benchmark label sets are not the actual label sets, they reflect our best knowledge of the cell types

	Dataset	$\#$Cells	$\#$Genes	$\#$Benchmark main-types (subtypes)	Protocols	Reference
Experimental	CT26.WT	9621	11,710	1(-)	10X v3	-
datasets	T-CELL	2989	7893	5(-)	10X	[24]
	CORTEX	7390	12,887	8(33)	inDrop	[25]
	RETINA	26,830	13,118	6(18)	Drop-seq	[26]
	IPF	114,396	20,354	5(31)	10X	[27]
	COVID	1,251,200	23,491	31(57)	10X	[28–30]
Simulated	SD1	4000	10,000	10(-)	-	-
datasets	SD2	4200	10,000	4(-)	-	-
	SD3	2800	10,000	2(4)	-	-
	SD4	3000	4887	5(-)	-	-

#### UMI counts of monoclonal cells follow gene-specific negative binomial (NB) distributions

The monoclonal cell population, CT26.WT, is a murine colon carcinoma cell line derived through monoclonal expansion (5: 5.4). We evaluated the “goodness-of-fit” of the following four families of distributions on this dataset. They are all families of NB and zero-inflated NB (ZINB) distributions. Their mean parameter modeling is similar; the difference lies in their dispersion and zero-inflation parameter modeling. See Additional file 1: Note 1 for their mathematical forms. These models also adjust for the cell library size via a size factor $s_c$ (Additional file 1: Note 1).

- *Gene-common NB*: NB with gene-common dispersion parameters;
- *Gene-common ZINB*: ZINB with gene-common dispersion parameters;
- *Gene-specific NB*: NB with gene-specific dispersion parameters;
- *Gene-specific ZINB*: ZINB with gene-specific dispersion parameters.

We used Pearson’s chi-squared test [31] to evaluate the “goodness-of-fit” of the four distribution families to the actual UMI count distributions in CT26.WT (Additional file 1: Fig. S1A). With the type I error of $5\%$, the test rejected $34.3\%$ poorly fitted genes for the gene-common NB family and $34.5\%$ of for the gene-common ZINB family. The rejection rates are high, indicating that these distribution families do not fit the UMI count distributions. In contrast, when applied to the gene-specific NB and ZINB families, the “goodness-of-fit” tests only rejected $9.7\%$ and $6.1\%$ of poorly fitted genes, respectively. The rejection rates are not far from the preset type I error rate of $5\%$, suggesting an overall good fit of these models. The test results suggest that the well-fitted distribution family should include the gene-specific dispersion parameters.

On the other hand, including the zero-inflation parameters is unnecessary because the UMI counts do not contain excessive zeros compared with the negative binomial distributions. This still allows the UMI counts to have many zero observations. Many previous studies supported the conclusion. These studies checked the UMI distributions in multiple monoclonal datasets from multiple tissue types and different sequencing depths, and all found that the UMI counts were not zero-inflated [32–35]. Specifically, Svensson [33] used eight public datasets and concluded that high-throughput droplet-based methods that make use of UMI counts are not zero-inflated; Cao et al. [35] further clarified that UMI counts are not zero-inflated even for low-throughput plate-based methods. However, for those sequencing protocols with no UMI counts but only raw read counts with PCR replicates, the read counts are more zero-inflated than the negative binomial distribution. The PCR replicates elevated the non-zero read counts and distorted the distributions. Thus, our model and method do not apply to the raw read counts with the PCR replicates.

**Fig. 1:**
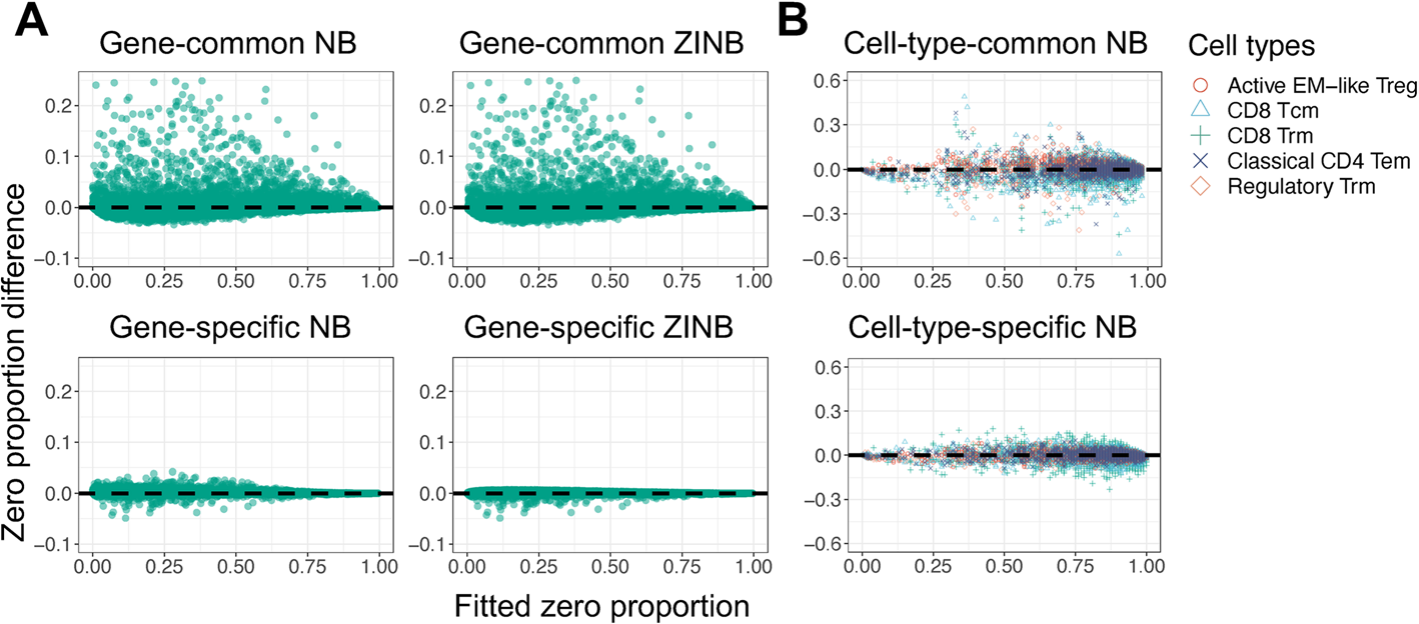
The fitting of six distribution families on the UMI count zero proportions. Six scatter plots show the difference between the observed and estimated UMI count zero proportions versus the fitted UMI count zero proportions. **A** Results for the monoclonal dataset, CT26.WT. Each dot represents a gene. The gene-common NB and ZINB families under-estimated the UMI zero proportions; the gene-specific NB and ZINB estimated zero proportions well. **B** Results for the polyclonal dataset, T-CELL. Each dot represents a gene in a benchmark cell type. The cell-type-specific NB family estimated the UMI count zero proportions better than cell-type common NB family

We split the CT26.WT into two datasets—half for training and the other half for testing. We derived the maximum likelihood estimators (MLEs) in each distribution family based on the training datasets and used the fitted distributions to estimate the zero proportions in the test dataset. When we compared the estimated and the actual UMI count zero proportions (Fig. 1A), we found that the gene-common NB and ZINB families underestimated the zero UMI count proportions in CT26.WT; in contrast, the gene-specific NB and ZINB families yielded reasonable and similar estimates of the zero UMI count proportions. Thus, adding zero-inflation parameters does not further improve fitting. Our results are consistent with the previous studies.

The difference between our results and the previous results is in choosing the proper NB family. Kim, Zhou, and Chen claimed that Poisson distributions are sufficient to model the UMI counts for most of the genes with only a few exceptions [34]. Townes et al. modeled the distributions of cellular gene UMI counts as an over-dispersed Dirichlet-multinomial distribution, which can be approximated by the independent NB family with gene-common dispersion parameters [32]. Svensson proposed to use the NB family with gene-common and cell-type-common dispersion parameters [33]. We evaluated the NB family with gene-specific mean and dispersion parameters and verified that it fits the monoclonal cell population better than the gene-common NB family. We also used the training and test split testing method to ensure this better fitting is not due to overfitting.

The gene-specific NB family is only designed to fit the raw UMI counts of the monoclonal cells; it fits poorly on the distribution of the normalized counts (FPKM in Additional file 1: Fig. S1B, TPM in Additional file 1: Fig. S1C). When the test type I error is $5\%$, the gene-common NB, gene-common ZINB, and gene-specific NB families do not fit the normalized count distributions for almost all genes; the gene-specific ZINB family does not fit the normalized count distributions for more than $30\%$ of the genes.

#### UMI count of polyclonal cells follow gene-specific cell-type-specific NB distributions

Polyclonal cell populations consist of cells from multiple cell types. It is possible that the UMI count distributions differ across cell types. Thus, we consider two NB distribution families: cell-type-common and cell-type-specific NB models. Here, the cell types refer to the actual cell types of the cell population. The difference between these two models is whether the mean and dispersion parameters are the same across cell types. See Additional file 1: Note 1 for their mathematical forms. Notably, these models have adjusted for the cell library size.

- *Cell-type-common NB*: NB with the cell-type-common but gene-specific mean and dispersion parameters;
- *Cell-type-specific NB*: NB with the cell-type-specific and gene-specific mean and dispersion parameters.

First, we explored which family fits the observed UMI counts in T-CELL better. T-CELL has a high-quality benchmark label set. Thus, we treated the benchmark cell types as the surrogates of the actual cell types. By using the cell-type-specific “goodness-of-fit” tests (5: 5.3), we found that the cell-type-specific NB family is better (Additional file 1: Fig. S2): with the type I error of $5\%$, the tests rejected $34.27\%$ of the genes for the cell-type-common NB family and $2.27\%$ for the cell-type-specific NB family. Thus, adding cell-type-specific parameters substantially improved the fitting to the UMI count distributions.

The cell-type-specific NB family has more degrees of freedom. To show that the improved fit does not stem from overfitting, we split the T-CELL dataset with half training and half testing samples. In the training samples, we fitted MLE to find parameters of each gene and each benchmarked cell type and then estimated the zero UMI count proportions on the test samples. When we compared the estimated and observed zero UMI count proportions in the test samples, we found that the cell-type-specific NB family provides much better estimates of the zero UMI count proportions (Fig. 1B). We performed similar analyses on the CORTEX and RETINA datasets and observed similar results (Additional file 1: Fig. S3–S6). These results show that the cell-type-specific NB family fits the UMI count distribution well.

### CDI overview

**Fig. 2:**
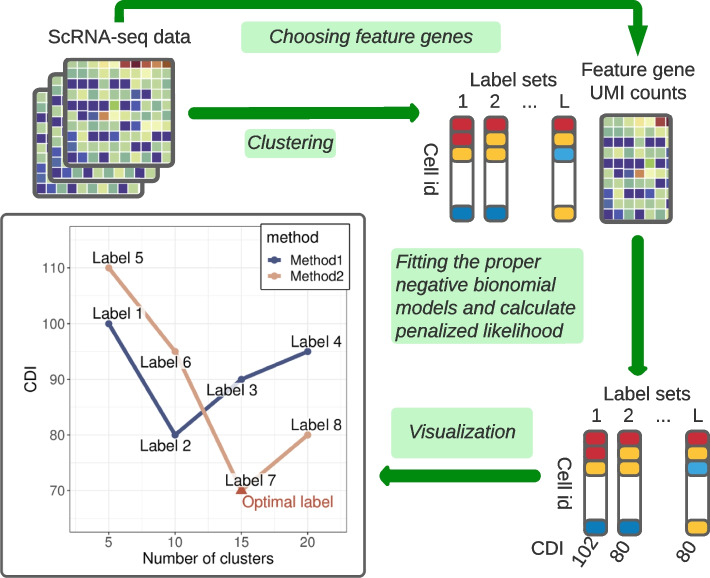
CDI flowchart. A graphical illustration for CDI

We developed CDI, an internal index, to evaluate the deviation between the observed and fitted UMI count distribution based on the candidate label set. For a given scRNA-seq dataset, first, we performed (a) feature gene selection procedure to derive low-dimensional feature genes and (b) various clustering methods with a wide range of tuning parameters to get candidate label sets. Second, we estimated the MLEs of the single-batch or multi-batch UMI count distributions based on each candidate label set and feature gene UMI counts. Third, we applied the AIC or BIC criterion to get the CDI index score for each candidate label set. The candidate label set with the lowest CDI index score is optimal. Because BIC puts a higher penalty on model complexity, CDI with BIC (CDI-BIC) favors label sets with fewer clusters. Thus, we recommend using CDI-BIC to select the optimal label set on main cell types. Conversely, we recommend using CDI with AIC (CDI-AIC) to select the optimal subtype label set to depict the heterogeneity with a higher resolution. See Fig. 2 for an illustration. See 5: 5.2 for more details.

CDI calculations are based on feature genes. Feature genes are those differentially expressed across cell types. Therefore, many scRNA-seq clustering methods rely on feature genes to cluster cells: selecting feature genes could substantially reduce data dimensions and possibly boost the signal in clustering. We also selected feature genes before calculating CDIs because of similar reasons. Many existing feature gene selection methods are available [8, 32, 36]. We developed a new approach using a working dispersion score (WDS). The working dispersion is the overall sample dispersion as if the UMI counts among all cells follow a common NB distribution. See 5: 5.1. WDS can capture the fold change of mean parameters across cell types (Additional file 1: Note 2). WDS selects the feature genes in the following way. For single-batch datasets, it selects the genes with the highest average WDS scores. For multi-batch datasets, it ranks the genes in each batch by their average WDS scores, takes the genes’ minimum rankings across batches, and selects the genes with the smallest minimum rankings. In 2: 2.3, we showed that CDI following WDS can select feature genes to distinguish different cell types under most circumstances.

### Performance evaluation

We evaluated the performance of WDS and CDI on four simulated datasets (SD1–SD4) and five experimental datasets (T-CELL, CORTEX, RETINA, IPF, and COVID).

All datasets have benchmark label sets. All simulated datasets use their true label sets as the benchmark label sets. For three moderate-scale datasets, T-CELL (2989 cells), CORTEX (7390 cells), and RETINA (26,830 cells), the benchmark label sets were obtained by the experimental and bioinformatics analysis process, including fluorescence-activated cell sorting (FACS), known feature gene checking, cell screening, and clustering (5: 5.4). These benchmark label sets are of high quality: although they are not the actual label sets, they reflect our best knowledge. For the two large-scale datasets, IPF (114,696 cells) and COVID (1,251,200 cells), annotating cells is very challenging. The large-scale cell population is often highly heterogeneous with hierarchical cell structures; thus, generating reliable clustering results is difficult. Also, traditional verification approaches like manually checking gene markers is too laborious for large-scale datasets. Thus, the benchmark label sets might not be as reliable. See more details in 2: 2.3.

To generate candidate label sets, we used multiple clustering methods for most datasets, each with a wide range of tuning parameters. These methods include hierarchical clustering, K-means clustering, spectral clustering, CIDR [5], Seurat v3 [8], and an ensemble clustering method called SC3 [10] (5: 5.5). For the large-scale datasets (IPF and COVID), we used Seurat v3 only because it is the most computationally efficient clustering method. For example, for IPF, Seurat v3 generated one clustering label set in around 30 mins; other methods cannot complete computing one candidate label set in 5 hours. Although we only applied Seurat v3 on IPF and COVID, we still applied a wide range of tuning parameters to generate 20 and 17 candidate label sets for IPF and COVID, respectively.

#### Performance of WDS

**Fig. 3:**
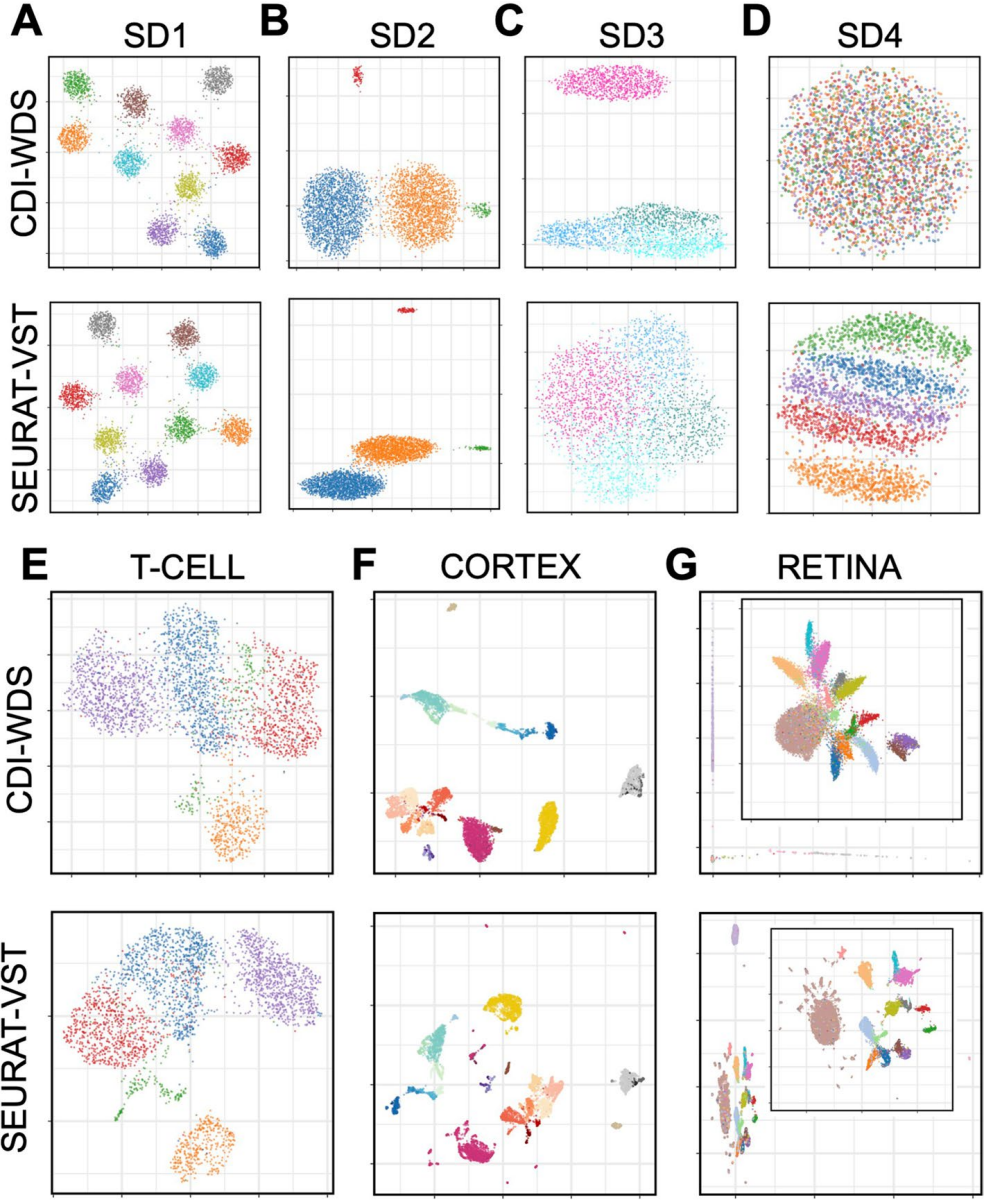
Comparisons between WDS and VST. **A**–**D** UMAPs of the simulated datasets (SD1–SD4). **E**–**G** UMAPs of the experimental datasets (T-CELL, CORTEX, and RETINA). In all plots, cells from different cell types are marked with different colors. Each panel contains two figures. Feature genes were selected by WDS in the first figure and by VST in the second figure

We compared WDS against another feature selection method, VST, the default for Seurat v3 [37] and v4 [38]. First, we selected the top 500 feature genes using WDS and VST, respectively. Second, we normalized the UMI counts of the selected feature genes by the $\log (\max (\text {count},0.1))$ transformation. Third, we calculated the top 50 principal components (PCs) of the normalized UMI counts. Finally, we plotted the two-dimensional uniform manifold approximation and projection (UMAP) [39] based on the top 50 PCs (Fig. 3).

- For all datasets except for SD3 and SD4, the UMAPs based on both the WDS-selected feature genes and the VST-selected feature genes separate different cell clusters well.
- For SD3, WDS selected 74/75 of the actual feature genes while VST only selected 48/75. Most of the feature genes missed by VST and found by WDS have high expression in three similar subtypes but low expression in the less similar main types. Consequently, the UMAP based on the WDS-selected feature genes reflects the cell type structure better than the VST-selected feature genes.
- For SD4, VST performed better than WDS. SD4 was generated from Splatter [40], a scRNA-seq data simulator that imposes strong mean-dispersion trends on gene expressions—that is, highly expressed genes are forced to have a lower dispersion. Such trends are commonly seen in bulk RNA-seq data, but we did not observe them in the UMI counts of the scRNA-seq datasets (Additional file 1: Fig. S7A, B). For datasets with such trends, WDS will select the genes with the lower average UMI counts. These genes contain little information on cell types; thus, the resulting UMAP cannot separate the cells from different cell types. Because splatter is a commonly used scRNA-seq data simulator, we included SD4 to check the robustness of the subsequent procedure of CDI. In practice, when such mean-dispersion trends exist for the UMI counts, we should not use WDS to select feature genes; however, when such mean-dispersion trends do not exist (as in all of our experimental datasets), WDS works well.

**Fig. 4:**
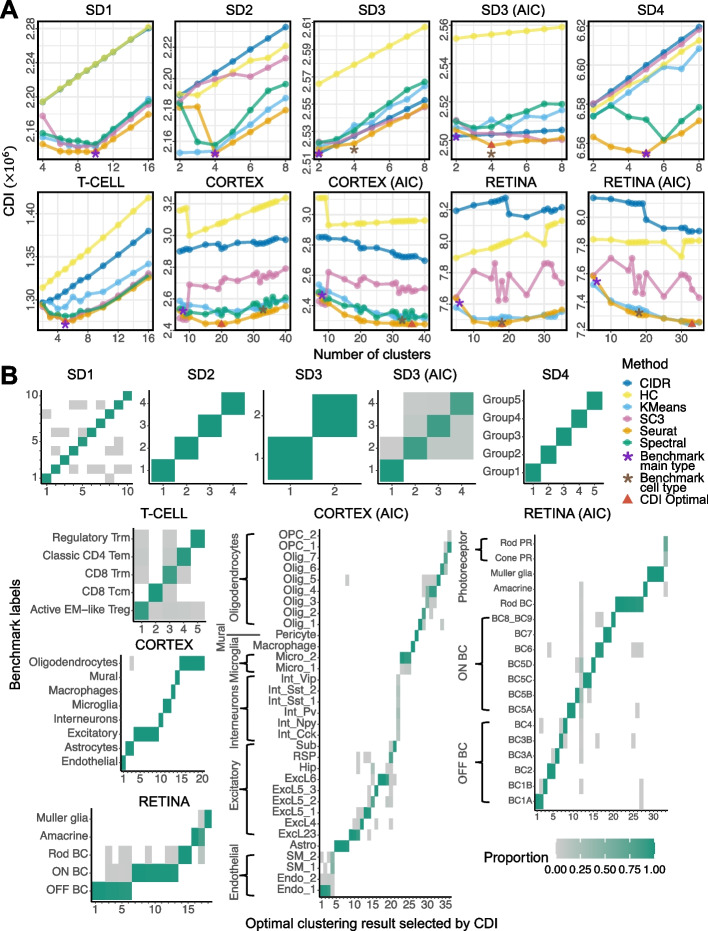
CDI’s performance. CDI was applied to four simulated datasets (SD1-SD4) and three experimental datasets (T-CELL, CORTEX, RETINA); all have high-quality benchmark label sets. CDI-AIC was applied to the panels with (AIC) in the subtitles; CDI-BIC was applied to the rest of the panels. **A** CDIs of the candidate label sets. The *x*-axis labels the cluster numbers, and the *y*-axis labels the CDI scores. For the datasets with hierarchical structures (SD3, CORTEX, and RETINA), both CDI-BIC and CDI-AIC were applied. The line color represents the clustering method. The red triangle marks the CDI-selected label set; the purple star marks the benchmark main type label set; the brown star marks the benchmark subtype label set. **B** The heatmaps show the cell proportions from the benchmark label sets for each cluster in the selected label sets. The *x*-axis labels the CDI-selected label set clusters, and the *y*-axis labels the benchmark cell types. The color of each rectangle represents the benchmark cell-type proportions in each selected label set cluster. Each column adds up to 1. PR: photoreceptor

When WDS and VST select different feature gene sets, even if their resulting UMAPs separate cell types similarly well, CDI based on the two gene sets could select different label sets. For example, for T-CELL, both UMAPs look similar (Fig. 3E). However, CDI following VST selected the sixteen-cluster label set generated by the spectral clustering with ARI 0.39; CDI following WDS selected the five-cluster label set generated by Seurat with ARI 0.87 (Additional file 1: Fig. S8). For reference, T-CELL’s benchmark label set has five clusters, similar to the five-cluster label selected by CDI following WDS (T-CELL panel in Fig. 4A, B). Thus, CDI following WDS is more robust and accurate.

When WDS selects different numbers of feature genes, CDI based on these different feature gene sets has robust performance. For example, for T-CELL, based on the 200 WDS-selected feature genes, CDI selected a six-cluster label set generated by Seurat; the second-best was the five-cluster label set generated by Seurat. Based on the 300 WDS-selected feature genes, CDI selected the five-cluster label set generated by Seurat. Finally, based on the 400 or 500 WDS-selected feature genes, CDI selected the five-cluster label set generated by SC3. (Additional file 1: Fig. S9). These label sets were similar to the benchmark label set (ARIs between 0.80 and 0.87). Thus, CDI’s performance is robust to the number of WDS-selected feature genes.

#### Performance of CDI

*A. Data containing no rare cell types.* We define a cell type as *rare* if its proportion is below $3\%$. We evaluated the performance of CDI on the datasets where none of the cell types are rare. These datasets include SD1, SD4, and T-CELL. SD1 contains ten equally proportional cell types simulated from the verified NB model; SD4 contains five unequally proportional cell types simulated from Splatter; T-CELL contains a mixture of five types of T cells. We selected their feature genes with either WDS (SD1, T-CELL) or VST (SD4) and then applied CDI-BIC to select the optimal label set marking their main cell types. The result shows CDI-BIC selected the label sets with the correct numbers of clusters for SD1, SD4, and T-CELL (SD1, SD4, T-CELL panels in Fig. 4A); moreover, the selected label sets are similar to the benchmark label sets (SD1, SD4, T-CELL panels in Fig. 4B). For SD4, CDI selected the label set identical to the benchmark label set; for SD1 and T-CELL, CDI selected the label set with $98\%$ and $95\%$ of the cells correctly allocating to their benchmark clusters. Notably, some candidate label sets have the correct numbers of clusters, but their cell labels are very different from the benchmarks. For example, for T-CELL, the two label sets with the lowest CDI are the five-cluster label sets generated by Seurat and SC3. These two label sets have similarly low CDIs ($1.2744\times 10^6$ for Seurat and $1.2743\times 10^6$ for SC3) and similarly high ARIs (0.875 for Seurat and 0.870 for SC3). However, the five-cluster label sets generated by other methods have much higher CDIs (1.3356$\times 10^6$, 1.3095$\times 10^6$, 1.2918$\times 10^6$, 1.2812$\times 10^6$) and much lower ARIs (0.002, 0.116, 0.132, and 0.388). Thus, the label sets with lower CDIs have higher ARIs. The heatmaps also verified that the label sets with lower CDIs are more similar to the benchmark label set (Additional file 1: Fig. S10). These results suggest that CDI has a similar performance to ARI in selecting the optimal label set when the data contain no rare cell types. Moreover, CDI has a significant advantage over ARI because its calculation does not rely on the benchmark label set.

*B. Data containing rare cell types.* We evaluated the ability of CDI-AIC and CDI-BIC to detect rare cell types. For example, SD2 simulated a cell population with two abundant ($47.62\%$ of all cells each) and two rare ($2.38\%$ of all cells each) cell types. Two abundant cell types and one rare cell types have different but similar feature gene UMI count distributions. This rare cell type is called RC1; the other rare cell type is called RC2 (Fig. 3A SD2 panel). For SD2, CDI-BIC selected a label set similar to the benchmark, suggesting that it can differentiate both rare cell types. Next, we reduced the cell proportion of RC1 further to challenge CDI with more complex tasks. When the cell proportion of RC1 reduced to $2.03\%$ (85/4185), CDI-BIC selected the three-cluster label set generated by spectral clustering; however, CDI-AIC still selected the four-cluster label set, including RC1 (Additional file 1: Fig. S11B, S11D). When the cell proportion of RC1 reduced to $0.49\% (20/4120)$, neither CDI-AIC nor CDI-BIC distinguished RC1; instead, they both selected the three-cluster label set generated by Seurat. Although this label set misses RC1, it has a very high ARI (0.98) (Additional file 1: Fig. S11G, S11I). Also, if we put the benchmark label set in the candidate pool, CDI-BIC would rank it as the fourth among all label sets, and CDI-AIC would select it as the optimal (Additional file 1: Fig. S11I). These results suggest that both CDI-BIC and CDI-AIC were able to detect rare cell types; compared with CDI-BIC, CDI-AIC is more sensitive in detecting rare cell types.

*C. Data with hierarchical cell type structures.* In scRNA-seq data, some main cell types can be further divided into subtypes. For example, SD3 simulated a cell population with two main cell types; one main cell type contains three subtypes, and the other is homogeneous. We used CDI-BIC to select the main type label set and CDI-AIC to select the subtype label set. As a result, CDI-BIC selected the two-cluster label set similar to the benchmark main type label set; CDI-AIC selected the four-cluster label set similar to the benchmark subtype label set (Fig. 4A SD3 panel, 4B SD3 panel). Another dataset, CORTEX, has eight main types and 33 subtypes. CDI-BIC selected a 20-cluster label set (Fig. 4A CORTEX panel). These 20 clusters correspond to the partitions of eight main types in the benchmark label set: clusters 4-10 correspond to excitatory neurons, clusters 11 and 12 correspond to microglia cells, and clusters 15–20 correspond to oligodendrocytes (Fig. 4B CORTEX panel). CDI-AIC selected a label set with 36 clusters (Fig. 4A CORTEX (AIC) panel). Some of these 36 clusters were partitions of the benchmark subtypes: they further partitioned endothelial cells subtype 1, astrocytes, excitatory cells subtype 23, excitatory neuron 5_1, excitatory neuron 6, oligodendrocytes subtype 5, and microglia subtype 2 into 21 clusters. Other clusters in the 36-cluster label set were mixtures of rare cell types, including a cluster mixing all interneuron subtypes (taking up $1.8\%$ of all cells), a cluster mixing two endothelial subtypes, and a cluster mixing two microglia subtypes (Fig. 4B CORTEX (AIC) panel). None of the candidate label sets can separate these rare cell types. These results suggest that when the data have hierarchical structures, applying CDI-BIC in combination with CDI-AIC is an excellent strategy to reveal its hierarchical structure. When the subtypes contain too few cells, CDI-AIC may fail to identify the rare subtype but can still cluster them with other similar subtypes.

*D. Data from multiple batches.* RETINA had two batches with six main types. Among them, photoreceptors were further divided into rod photoreceptors ($0.34\%$) and core photoreceptors ($0.18\%$. The ON cone bipolar cells (BCs) had seven subtypes; the OFF cone BCs had six. CDI-BIC selected an 18-cluster label set that classified the main types well. It partitioned the rob BCs, ON cone BCs, and OFF BCs into several subtypes. On the other hand, CDI-AIC selected a label set with 33 clusters. This label set separated all the subtypes well. Besides, it provided a more exemplary partition on the benchmark Müller glia cells and the rod bipolar cells (RBCs). Some subtypes of ON cone BCs (BC5A, BC5C, BC6, BC7) and OFF cone BCs (BC1A, BC2) were also further partitioned into sub-clusters. Cluster 11 spanned many cell types; however, they are mainly BCs. These results suggest that CDI works well on the multi-batch scRNA-seq dataset.

*E. Large-scale datasets.* IPF (114,696 cells) and COVID (1,251,200 cells) are two large-scale datasets. For IPF, the benchmark label set contains 31 clusters. This is a low-resolution label set. For example, the benchmark microphage group contains 38,928 cells; they were not further divided. We applied Seurat v3 to generate 20 candidate label sets with cluster numbers ranging from 5 to 100. Then, we applied CDI-BIC to select the optimal label set, which contains 80 clusters (Additional file 1: Fig. S12). The selected label set has many more clusters than the benchmark label set. In particular, it further divided the benchmark AT2, ciliated, endothelial, macrophage, T, and MUC5B$+$ cells into multiple subpopulations (Additional file 1: Fig. S13). All the benchmark cell types contain a few outliers far from most other cells of the same cell type (Additional file 1: Fig. S14). Also, macrophages, endothelial, and MUC5B$+$ cells exhibit apparent heterogeneity. This implies that the benchmark label set might not be accurate enough. In general, annotating large-scale scRNA-seq datasets is challenging. CDI provides an effective way to assist this task. The benchmark label set for another large-scale dataset, COVID, has three layers of hierarchical annotations. The quality of the benchmark label set is also limited (Additional file 1: Fig. S15). For example, layer 2 CD4 TCM and TEM cells contain the cells marked as CD8 T cells based on layer 3 annotations; layer 2 B memory and naive cells also contain the cells marked as CD4 and CD8 T cells based on layer 3 annotations. Also, even on the layer 3 annotations with the highest resolution, the CD14 monocytes still contain 312,430 cells. They are unlikely to be homogeneous. Due to the low quality of its benchmark annotations, we did not compare the selected label set (Additional file 1: Fig. S16) with the benchmark annotation. However, these datasets were still useful for evaluating the computation time evaluation of various indices. See 2: 2.3.

#### Comparison of CDI with other internal indices

For general clustering problems, many other internal indices have been developed, including the Calinski-Harabasz index [14], Connectivity [15], Davies-Bouldin index [16], Dunn index [17], Baker-Hubert Gamma index [18], SD index [19], Silhouette index [41], and Xie-Beni index [20]. Details of these eight internal indices are listed in Table 2. Although these indices are not customized for scRNA-seq data clustering, they have been used to select scRNA-seq clustering label sets [42–44]. We compared the performance of CDI with these internal indices based on their performance on the four simulated datasets (SD1–SD4) and three experimental datasets (T-CELL, CORTEX, and RETINA). We did not include IPF and COVID in the comparison because they do not have high-quality benchmark label sets. More important, CDI is the only computationally feasible method for COVID; other internal indices failed on such large-scale datasets. See 2: 2.3.

**Table 2:** Summary of three external indices and eight internal indices

	Index name (abbreviation)	Method	Optimal value	Reference
External	Adjusted Rand Index (ARI)	Rand index adjusted for chance	Max ($=1$)	[11]
indices	Fowlkes-Mallows index (FM)	Sensitivity and precision	Max ($=1$)	[12]
	Normalized Mutual Information (NMI)	Information theory	Max ($=1$)	[13]
Internal	Calinski-Harabasz index (CH)	Within/between-cluster variance	Max	[14]
indices	Connectivity index (Connectivity)	Clustering consensus of nearest neighbors	Min	[15]
	Davies-Bouldin index (DB)	Within/between-cluster distance	Min	[16]
	Dunn index (Dunn)	Within/between-cluster distance	Max	[17]
	Baker-Hubert Gamma index (Gamma)	Within/between-cluster distance	Max	[18]
	SD index with average scattering (SD-Scat)	Within/between-cluster variance	Min	[19]
	Silhouette index (Silhouette)	Within/between-cluster distance	Max	[41]
	Xie-Beni index (XB)	Within/between-cluster distance	Min	[20]

**Fig. 5:**
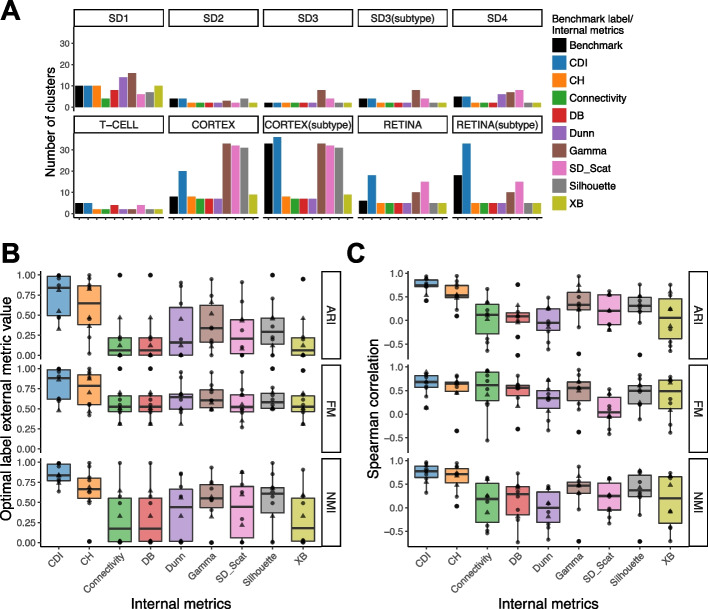
Comparison between CDI and other internal indices. **A** The barplot shows the cluster number of the benchmark label set and the label sets selected by different internal indices. Each panel compares the cluster number of a benchmark label set with all other selected label sets for one dataset. For the dataset with both main type and subtype benchmark label sets (SD3, CORTEX, and RETINA), the panel with the subtype benchmark label set is listed with “(subtype)”. **B** The boxplot shows the external indices of the label sets selected by different internal indices. The external indices are calculated by comparing the selected label sets and the benchmark label sets. **C** The boxplot shows the Spearman correlation between the internal and external indices while ranking all candidate label sets. The dots in **B** and **C** mark the external indices or Spearman correlations for main type benchmark label sets. The triangles mark the external indices or Spearman correlations for subtype benchmark label sets

We checked the cluster numbers in the optimal label sets selected by the nine internal indices (Fig. 5A). For all simulated datasets (SD1–SD4) and T-CELL, the CDI-selected label set has the same number of clusters as the benchmark label set. Other internal indices did not select the same number of cell types as the benchmark label set: CH, Connectivity, DB, Silhouette, and XB often select the label set with fewer clusters; Gamma usually selects the label set with more clusters; Dunn and SD-Scat sometimes select more clusters and sometimes select fewer clusters. For CORTEX and RETINA, the CDI-selected label sets have more clusters than the benchmark label set: the selected label set is similar to a finer partition of the benchmark label set. CDI-AIC selected the label set with a similar cluster number to the CORTEX subtype benchmark label set. All other internal indices only select one optimal label set. For CH, Connectivity, DB, Dunn, and XB, the selected label sets have similar cluster numbers as the main type benchmark label sets; for Gamma, SD-Scat, and Silhouette, the selected label sets have similar cluster numbers as the subtype benchmark label sets.

However, the number of clusters the selected label sets do not fully reflect the quality of the selected label set. To evaluate the quality of the optimal label set selected by various internal indices, we compared these label sets with the benchmark label sets using external indices: ARI, FM, and NMI (Table 2). If the selected label set is more similar to the benchmark label set, it will have higher ARI, FM, and NMI. In Fig. 5B, each dot shows the external index score between each selected label set and the main type benchmark label set; each triangle shows the external index score between each selected label set and the subtype benchmark label set. For most datasets, the CDI-selected label sets have higher ARI, FM, and NMI and thus are more similar to the benchmark label sets. The median scores of the CDI-selected label sets are also the highest for all ARI, FM, and NMI. This suggests CDI selected label sets closest to benchmark label sets in terms of external metrics.

In addition to checking the selected label set, we evaluated the performance of the internal indices on all candidate label sets. An internal index is better if it ranks all candidate label sets more similarly than the external indices. Thus, we quantified the Spearman correlation [45] between the internal and external indices. See 5: 5.6 for details. Since five internal metrics (CDI, Connectivity, Davies-Bouldin index, SD index, and Xie-Beni index) mark better label sets with smaller scores, we reversed their rankings when calculating their Spearman correlations with external indices. Then, the best internal index should have the highest Spearman correlation. Figure 5C marked the Spearman correlation for the main type benchmark label sets as dots and the subtype benchmark label sets as triangles. CDI consistently has a higher median Spearman correlation with all three external indices, ARI, FM, and NMI. For example, the median Spearman correlation of CDI and ARI is 0.74, greater than the second-best performer, CH, whose Spearman correlation with ARI is 0.53. This suggests that CDI has the highest agreement in ranking candidate label sets with the external indices and thus outperforms all other indices in scoring label sets. Moreover, we found that the Spearman correlations have the smallest interquartile range between CDI and ARI/NMI and the third smallest interquartile between CDI and FM. This suggests that CDI is robust across datasets.

#### Computation time

**Fig. 6:**
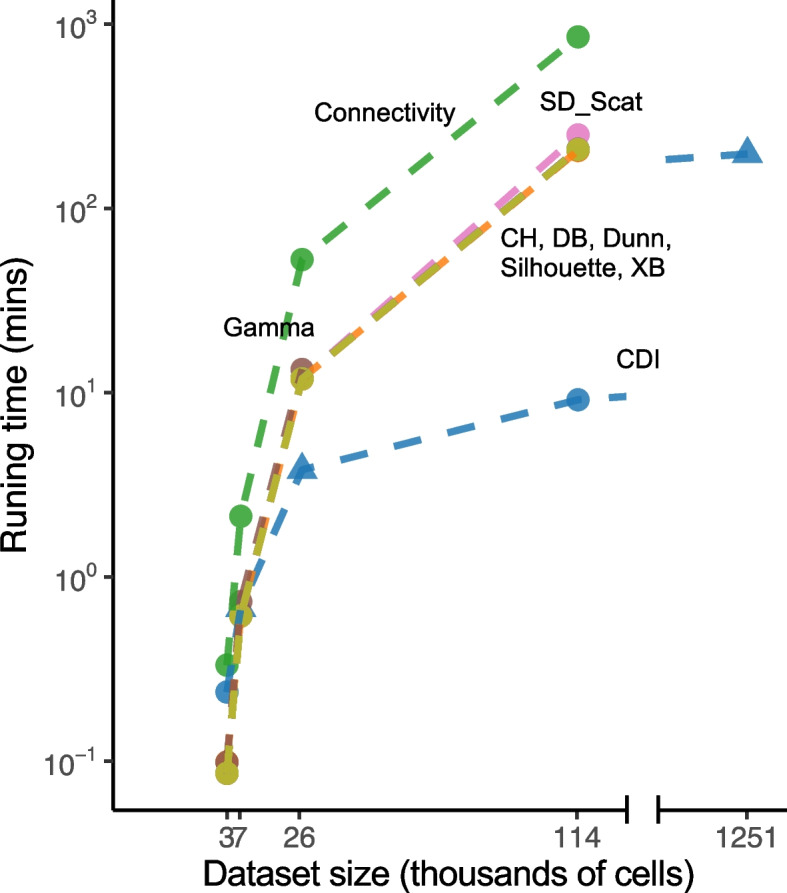
Computation time of nine internal indices. The computation time of all internal indices was evaluated on the five experimental datasets (Table 1) based on 500 selected genes and benchmark cell type labels. The *x*-axis labels the cell numbers in thousands of these datasets. CDI allows considering batch effect. The triangle corresponds to CDI calculation considering the batch effect, and the dot corresponds to calculating CDI without batch effect. The *y*-axis labels the computation time in the log10 scale. Algorithms of indices were tested on one Intel(R) Xeon(R) Gold 6252 CPU

We compared the computation time for nine internal indices, including CDI (Fig. 6).  CDI was computed by our R package CDI[46], Connectivity was computed by the R package clValid [47], and all other internal indices were computed by the R package clusterCrit [48].

The computation time was evaluated on the benchmark label set of the five experimental datasets (Table 1). For T-CELL (2,989 cells), all internal indices took less than one minute. For CORTEX, Connectivity took 2.13 min; all other indices took less than 1 min. For RETINA with 26,830 cells, CDI took only 3.81 min, five internal indices (CH, DB, Dunn, SD-Scat, Silhouette, XB) took about 12 min, Gamma took 13.96 min, and Connectivity took 52.77 min. CDI was at least three times faster than all other indices. For IPF with 114,396 cells, CDI has a more obvious advantage in computation time: CDI took only 9.15 min; in contrast, five indices (CH, DB, Dunn, Silhouette, XB) took $206\sim 210$ min, SD-Scat took 250.56 min, and Connectivity took 853.01 min. Another internal index, Gamma, failed to output the index score for IPF because its function in the clValid package cannot handle such large cell numbers. For COVID with 1,251,200 cells, CDI took 197.47 min. It was the only internal index that was computationally feasible for such a large-scale dataset. Some internal indices (CH, Connectivity, Gamma, Silhouette, XB) cannot handle such large cell numbers because of memory issues. These indices are based on the pairwise distance matrices among cells. For a large dataset like COVID, computing and storing a 1.2 million times 1.2 million matrix takes too much memory. Other indices (DB, Dunn, SD-Scat) cannot finish within 24 h, so we stopped the computation. Noteworthily, the above computation time is only for computing one label set. We recommend computing the index scores on at least ten label sets to select the optimal label set; thus, the overall time is even longer.

Moreover, CDI’s computation time was measured in a single-core machine on all datasets for a fair comparison with other internal indices. However, CDI also allows for parallel computing across genes; thus, the computation time could be reduced tremendously.

Finally, CDI’s computation time does not depend on the number of clusters. When we evaluated CDI’s computation times on 20 IPF candidate label sets whose cluster numbers range from 5 to 100, CDI’s average computation time was 10.26 min with a standard deviation of 0.29 min. For single-batch datasets, CDI’s computational cost is mainly from estimating the maximum likelihood, and its time complexity is *O*(*GN*), where *G* denotes the number of feature genes, and *N* denotes the number of cells. For multi-bath datasets, the additional batch-effect testing procedure has complexity *O*(*BGN*), where *B* is the number of batches.

## Discussion

We developed a new index, CDI, to calculate the deviation between the candidate label set and the observed UMI counts. CDI calculates the negative penalized maximum log-likelihood of the feature gene UMI counts for each candidate label set. The likelihood function is based on the gene-specific cell-type-specific NB distribution family verified in the monoclonal and polyclonal scRNA-seq datasets. We recommend using WDS to select the feature genes for CDI because CDI following WDS is more robust than VST for this purpose.

Because calculating CDI relies on the gene-specific cell-type-specific NB distribution family, we would like to elaborate on two major innovations of our approach to ensure the distribution family is reliable.

First, monoclonal single-cell datasets are essential to characterize the UMI count distributions in scRNA-seq data. With the well-characterized distributions, we can use the model validation tools such as AIC or BIC to evaluate the deviation from the data to the model given the candidate label set. To generate a monoclonal dataset, we cloned a single mother cell to derive a cell line whose components can be considered identical. Previously, such monoclonal datasets were generated by either spiking in the ERCC RNA or purifying cells by FACS. The ERCC RNA samples contain the synthesized RNAs differing from the endogenous transcripts in many aspects [23, 49] (such as length, guanine-cytosine content, 5′ cap, polyA length, and ribosome binding). These structural disparities lead to different conversion efficiencies of mRNA into cDNA. Thus, while ERCC eliminates the cell type variations, they also eliminate or distort the variations in library construction, sequencing depths, and cell cycles. Another choice, FACS, can keep these variations; however, it only purifies cells based on a limited number of protein markers and therefore can only reduce but not eliminate the cell type heterogeneity in a cell population. Unlike these two existing strategies, we used the single-cell expansion strategy to ensure an ideal monoclonal population: it keeps the variations in library construction, sequencing depths, and cell cycles; on the other hand, it also eliminates cell type heterogeneity.

Second, although others have suggested that the UMI count distributions are not necessarily zero-inflated, we use a new gene-specific and cell-type-specific models to characterize the UMI count distributions. Previous studies [32–34] used either Possion or gene-common dispersion NB distributions to model the scRNA-seq data. To derive a reliable NB distribution family, we used monoclonal and polyclonal datasets to evaluate multiple families of NB distributions. Finally, we found that the cell-type-specific and gene-specific NB distribution family fits the best.

Next, we would like to elaborate on CDI’s key features and limitations.

First, calculating CDI relies on the likelihood of the raw UMI counts. If only the normalized UMI counts are available, CDI cannot be applied.

Second, CDI is not a clustering method; instead, it is an index to evaluate the quality of the candidate label set: the label set with the lowest CDI will be selected. Thus, the quality of the selected label set highly depends on the quality of the candidate label sets. If none of the candidate labels fits the data well, the CDI-selected label set will not improve the fitting. Thus, providing a large pool of candidate label sets is crucial. We suggest applying CDI to at least ten candidate label sets to select a reliable label set.

Third, CDI-AIC puts fewer penalties on cluster numbers and usually selects the label with more clusters than CDI-BIC. These clusters often correspond to cell subtypes. Some of these cell subtypes could mark the cell transition stages. In many cases, the collected cells have different developmental stages so that the scRNA-seq data exhibit trajectory patterns. We can consider the cell types in those datasets as “continuous.” When discrete clustering methods are applied to these datasets, the resulting clusters often represent a local stage on the trajectory. Although CDI is not designed to select the optimal trajectory, we can use it to select the label sets corresponding to the optimal local stages. For example, in RETINA, the subtypes of ON cone BCs and OFF cone BCs represent different development stages of those BCs. The CDI-AIC successfully selected a satisfying discrete label set that approximates the continuous trajectories of ON cone BCs and OFF cone BCs (Fig. 4B. RETINA (AIC)).

In summary, finding an optimal clustering label set for scRNA-seq data is critical because clustering impacts all downstream analyses; CDI provides a robust and accurate internal method to select cluster label sets and leads to reliable downstream analysis.

## Conclusions

In this study, we developed a new internal index, CDI, to select the clustering label set that best fits the single-cell RNA-seq data among several candidates. The computation of CDI is easy and efficient. In both in silico and experimental studies, CDI successfully selected the biological meaningful clustering label set. The results of CDI are also stable and robust when we choose different numbers of feature genes or the data contain rare cell types or complex cell population structures. Unlike other general internal indices not designed for scRNA-seq data, CDI has a much better performance with respect to the benchmark labels and concordance with external indices. Although CDI does not require the knowledge of the benchmark label set, its performance is comparable with external indices such as ARI.

## Methods

### WDS

Before we apply CDI, we use WDS to select a set of feature genes containing cell type information. This is mainly for reducing dimensions for CDI calculation.

WDS is a score to measure the average sample (cell) dispersion of each gene.

1 $$\begin{aligned} \hat{\xi }_g = \frac{\hat{\sigma }_g^2-\hat{\mu }_g}{\hat{\mu }_g^2}, \end{aligned}$$

where $\hat{\mu }_g$ and $\hat{\sigma }_g^2$ are the sample mean and variance of gene *g*’s UMI counts in the pooled data. It can be viewed as the estimator of $\xi _g = (\sigma _g^2-\mu _g)/\mu _g^2$, where $\mu _g$ and $\sigma _g^2$ are the mean and variance of gene *g*’ UMI counts. The rationale of WDS is provided in the Additional file 1: Note 2.

Before selecting the feature genes, we filtered the genes with more than $95\%$ zero counts. We computed WDS for each gene and selected the top $G_1$ (default $G_1=500$) genes with the highest WDS. For datasets with multiple batches, we calculated and ranked WDS for genes in each batch: the minimum rank across the batches is set as the overall rank for each gene. In the end, we selected the top $G_1$ (default $G_1=500$) genes with the highest overall rank as the feature genes.

In the R package CDI, feature genes can also be added manually. For example, the users can use the feature genes provided by other approaches or domain knowledge.

### CDI

Suppose after we apply a clustering method to the scRNA-seq data, the resulting label set is $\varvec{L}=(L_1,\ldots ,L_N)'$ with $L_c\in [K]$, indicating the cell types of *K* clusters. This label set is not necessarily a good label set. We need to use CDI to evaluate the goodness-of-fit of this label set to the scRNA-seq data.

The key idea of CDI is that for any given label set, we pretend that it is the true label set and partition cells into subpopulations according to this label set. Within each partition and each gene, the cells share the same parameters. Then, we calculate the penalized negative log-likelihood based on this label set, called the CDI score. If the label set is close to the true label set, the CDI score is small; otherwise, it is large.

We used the cell-type-specific and gene-specific NB distribution to characterize the UMI counts of the selected feature genes. Specifically, for multi-batch scRNA-seq datasets, we also allow the existence of batch effects.

- For single-batch scRNA-seq dataset, the likelihood function is
  2 $$\begin{aligned} \ell (\mu _{gk}, \phi _{gk}: g\in [G], k\in [K]) = \sum _{g\in [G]} \sum _{c\in \mathcal {S}_b} \log \{F(X_{gc}\mid s_c\mu _{g L_c}, \phi _{g L_c})\}. \end{aligned}$$
  Here, *F* is the NB probability mass function (pmf) of $X_{gc}$, the UMI counts of feature gene *g* in cell type *k*. Specifically, $X_{gc}\mid (L_{0, c} = k) \sim \textrm{NB}(s_c\mu _{gk}, \phi _{gk})$, where $s_c$ is a scale factor to adjust for cellular library size (Additional file 1: Note 1), and $\mu _{gk}$ and $\phi _{gk}$ are the mean and dispersion parameters of gene *g* in cell type *k*. The mathematical form of the pmf is shown in Additional file 1: Note 1.
- For multi-batch scRNA-seq dataset, the likelihood function is
  3 $$\begin{aligned} \ell (\mu _{gk}^{(b)}, \phi _{gk}^{(b)}: g\in [G], k\in [K], b\in [B]){} & {} =\nonumber \\ {}{} & {} \sum _{b\in [B]} \sum _{g\in [G]} \sum _{c\in \mathcal {S}_b} \log \{F(X_{gc}\mid s_c\mu _{g L_c}^{(b)}, \phi _{g L_c}^{(b)})\}. \end{aligned}$$
  Here, *F* is the pmf of $X^{(b)}_{gc}$, the UMI counts of feature gene *g* in cell type *k* and batch *b*. Specifically, $X_{gc}^{(b)} \mid (L_{0,c}^{(b)}=k) \sim \textrm{NB}(s_c^{(b)}\mu _{gk}^{(b)}, \phi _{gk}^{(b)})$. It is likely that some gene *g* in cell type *k* does not have obvious batch effects so that its corresponding mean and dispersion parameters $\mu _{g L_c}^{(b)}$ and $\phi _{g L_c}^{(b)}$ are the same across batches. Thus, to better characterize the multi-batch data, we test if the batch effect is significant. For each gene *g* in each cell type *k*, we set the hypothesis
  $$\begin{aligned} \textrm{H}_{0,gk}: \ \mu _{gk}^{(1)}= \ldots = \mu _{gk}^{(B)}, \quad \phi _{gk}^{(1)}= \ldots = \phi _{gk}^{(B)}. \end{aligned}$$
  We used the likelihood ratio test to test this hypothesis. If the hypothesis is rejected, it indicates that the batch effect is significant for the gene in this cell type; thus we will introduce the batch-specific parameters. Otherwise, we will force same $\mu _{g L_c}^{(b)}$ and $\phi _{g L_c}^{(b)}$ across batches, *i.e.*, $\mu _{g L_c}^{(b)}=\mu _{g L_c}$ and $\phi _{g L_c}^{(b)} = \phi _{g L_c}$.

Next, we obtain the MLE of the mean and dispersion parameters of the likelihood functions. Denote the corresponding maximum likelihood by $\hat{\ell }$. To adjust for the model complexity, we use the penalized negative log-likelihood function as CDI

$$\begin{aligned} \tilde{\ell } = -2 \hat{\ell } + c_{\textrm{pen}} \cdot d. \end{aligned}$$

Here, $d = \sum _{g\in \mathcal {G}}\sum _{k\in \mathcal {K}} d_{gk}$ is the overall degree of freedom of the model. For a single-batch dataset or a multi-batch dataset with $\textrm{H}_{0,gk}$ accepted, $d_{gk} = 2$; for a multi-batch dataset with $\textrm{H}_{0,gk}$ rejected, $d_{gk}=2B$. $c_{\textrm{pen}}$ is the scale of penalty: $c_{\textrm{pen}}=2$ for AIC and $c_{\textrm{pen}}=\log (N)$ for BIC. Because BIC adds more penalty on the model degree of freedom, it prefers the models with fewer numbers of clusters. Thus, we use CDI-BIC to select the optimal main type label set and CDI-AIC to select the optimal subtype label set.

### Cell-type-specific “goodness-of-fit” tests

The cell-type-specific “goodness-of-fit” tests were performed on T-CELL, CORTEX, and RETINA. All three datasets have high-quality benchmark label sets. The testing process is as follows for each dataset.

- We filtered the genes and the cells as described in 5.4. Then, we performed the test on each gene after filtering.
- First, based on the benchmark label set, we assigned the cells into $5K_0$ bins, where 5 is the number of UMI count categories and $K_0$ is the number of the benchmark cell type. Based on the values of $(X_{gc}, L_{c})$, we assigned cell *c* into one of the following bins:
  $$\begin{aligned} \mathcal {U} \times \mathcal {K}, \quad \mathcal {U} = \{ \{0\},\{1\},\{2\}, \{3\}, [4,\infty )\}, \quad \mathcal {K} = \{1,\ldots ,K\}, \end{aligned}$$
  where $\times$ is the Cartesian product of two sets.
- Second, we computed the test statistic as
  $$\begin{aligned} T_{g} = \sum _{(U,k)\in \mathcal {U}\times \mathcal {K}}\frac{\left( n_{U,k} - N\hat{\pi }_{U,k} \right) ^2}{N\hat{\pi }_{U,k}}. \end{aligned}$$
  Here, $n_{U,k} = \sum _c I(X_{gc}\in \mathcal {U}, L_{c}=k)$, and $\pi _{U,k} = P(X_{gc}\in \mathcal {U}, L_{c}=k)$, which are the parameters of the multinomial distributions on $(n_{U,k}: U\in \mathcal {U}, k\in \mathcal {K})$. Because $\pi _{U,k}$ is unknown, we estimated $\pi _{U,k}$ by first expressing it as a function of $(\mu _{gk}, \phi _{gk})$ in the corresponding NB distribution family and then derived the maximum likelihood estimator (MLE) in the multinomial likelihood [31].
- Third, if $T_g$ is larger than the $95\%$ quantile of the chi-square distribution with the degree of freedom $5K_0-p-1$, we rejected the “goodness-of-fit” hypothesis for gene *g*. Here, *p* is the number of parameters in the corresponding NB distributions: for cell-type-common NB distributions, $p=2$; for cell-type-specific NB distributions, $p=2K_0$. We used the chi-square quantile as the threshold because when the UMI count of gene *g* follows the corresponding NB model and $L_c$ all match the true cell types, $T_g$ asymptotically follows $\chi ^2(5K_0-p-1)$ [31].
- Finally, we performed the test for all the genes and calculated the rejection proportion. We used the proportion as the criterion to assess the overall fitting of the corresponding NB distribution family and the UMI count distribution.

### Datasets

#### Simulated datasets

We simulated three sets of single-cell data (SD1-SD3) from the negative binomial distribution with gene-specific and cell-type-specific parameters. More specifically for SD1-SD3, the gene expression level for cells in cell type *k* and gene *g* were randomly sampled from NB$(\mu _{gk}, \phi _{gk})$, where $\mu _{gk}$ represented the mean parameter and $\phi _{gk}$ represented the dispersion parameter. Each dataset contained 10,000 genes, and the number of cells ranged from 2800 to 4200. To test the robustness of CDI, we generated SD4, which contained many outliers, and the UMI count distributions no longer followed the verified NB distribution.

1. We generated ten equal-sized cell groups. Each group contained 400 cells; thus, in total there are 4000 cells. One cell type was treated as the baseline type with $\mu _{gk}$ generated from the truncated normal distribution with mean 0.2 and standard deviation 0.1, and $\phi _{gk}$ generated from the truncated normal distribution with mean 0.5 and standard deviation 0.1. For each of the other nine groups, 25 genes had mean parameters shifted from the baseline group with log2 fold change 2.4. The feature gene dispersion parameters were shifted by a Gaussian-distributed factor with mean 0 and standard deviation 0.05.
2. We generated two abundant cell types with 2000 cells each and two rare cell types with 100 cells each. One abundant cell type was treated as the baseline group with $\mu _{gk}$ generated from the truncated normal distribution with mean 0.2 and standard deviation 0.1. The other abundant cell type contained 40 feature genes with log2 fold change of mean 1.5. One rare cell type, RC1, contained 50 feature genes with log2 fold change of mean 2.8; the other rare cell type, RC2, contained 50 feature genes with log2 fold change of mean 3.2. Because the log2 fold change of RC1 is smaller than RC2, RC1 is considered to be more similar to the two abundant cell types. The dispersion parameters $\phi _{gk}$ were set in the same way as in SD1.
3. We generated two main-types: C1 contains 1000 cells from a homogeneous cell type, and C2 contains 1800 cells from 3 subtypes. C1 is the baseline group with $\mu _{gk}$ generated from the truncated normal distribution with mean 0.4 and standard deviation 0.1 and $\phi _{gk}$ generated from the truncated normal distribution with mean 1 and standard deviation 0.1. Each subtype of C2 contains 600 cells and 40 feature genes. Among these 40 feature genes, 30 were shared by all subtypes, and the rest 10 were exclusive for each subtype. The log2 fold change in the means was 4 for the main type and 1.8 for the subtype. The dispersion parameters $\phi _{gk}$ were set in the same way as in SD1.
4. We generated five common cell types using the R package Splatter [40] with 3000 cells and 5000 genes. The probabilities that a cell belongs to any cell group were 0.2 for all groups. The proportion of differentially expressed genes were $1\%$ per cell group. The location and scale parameters of the log-normal distribution for these feature genes were (0.4, 0.1). In addition, we followed the default option to add $5\%$ of outliers. After we filtered the cells with less than $1\%$ non-zero counts and the genes with less than $1\%$ of non-zero cells, the dataset had 4887 remaining genes. See Additional file 1: Fig. S17 for all the parameters used in this setting.

#### Experimental datasets

CT26.WT.  The dataset was generated in Dr. Qi-Jing Li’s lab. Dr. Li is one of the co-authors of this paper. The wild-type CT26 cells from the murine colorectal carcinoma cell line were single-cell-diluted, and one clone was picked and cultured for 220 days. For the single-cell RNA-seq library preparation, 10, 000 cells of each clone were processed with the protocol of Chromium Single Cell 3′ Reagent kits v3 from 10X Genomics to make the single-cell RNA sequence library. Cells with more than $10\%$ mitochondrially derived transcripts were removed. Among these cells, we selected those with a non-zero gene proportion greater than $3\%$ or the number of non-zero genes greater than 300 (at least one of the two conditions needed to hold). We further selected genes with non-zero count proportions greater than $1\%$ or the number of non-zero cells greater than 50. This dataset was of high quality, with 24,208 median UMI counts per cell and 4376 median genes per cell. Since this dataset was highly homogenous, we used CT26.WT to evaluate the Pearson’s chi-squared “goodness-of-fit” of different models to the UMI counts in the monoclonal scRNA-seq data.
T-CELL. The T-CELL dataset was generated in our previous study [24]. The benchmark clustering labels of the T-CELL population were generated as a combination of protein-marker-based flow sorting labels and bioinformatics labels from Seurat v2. For evaluation purpose, we selected 5 distinct cell types: regulatory Trm cells, classical CD4 Tem cells, CD8 Trm cells, CD8 Tcm cells, and active EM-like Treg cells. In this study, tumors were firstly collected from the female mice after 3 weeks since the mice were injected by 4T1 tumors. Tissues were then disassociated into single cells and homogenized. T cells were separated out by flow sorting with a stringent gating threshold and sequenced on the 10X platform. For preprocessing, we filtered out genes with less than $2\%$ non-zero cells and removed cells with less than $2\%$ non-zero genes. Eventually, 2989 cells from five cell types with 7893 genes were retained.
CORTEX. The visual cortex dataset was generated by Hrvatin et al. [25] using inDrop to study the diversity of activity-dependent responses across cortical cell types. We obtained the labeled scRNA-seq dataset from [50], which contained 10,000 cells with 19,155 genes. Among these 10,000 cells, 7390 cells were identified to 33 cell types as an intersection of Seurat v1 and a density-based method [51]. In addition, eight main cell types (excitatory neurons, oligodendrocytes, astrocytes, interneurons, etc.) were annotated with known feature genes. We selected cells with at least 300 or $3\%$ of non-zero genes and genes with at least 50 or $1\%$ non-zero cells. After preprocessing, 7376 cells with 12,887 genes were included in clustering.
RETINA. The mouse retina dataset was generated using Drop-seq to classify retinal bipolar neurons [26]. This dataset contained 27,499 cells with 13,166 genes. Among these 27,499 cells, 26,830 cells were labeled with 18 cell types by the assembled pipeline: they first used Louvain-Jaccard [52] method to cluster the cells and then annotated the clusters with known feature genes. This 18-cluster label set was treated as the benchmark subtype label set. We further grouped the cell types into 6 main types based on the original paper. These cells came from two experimental batches of FAC sorted Vsx2-GFP positive cells on different days. These cells came from two experimental batches of FAC sorted Vsx2-GFP positive cells on different days. We selected the cells with at least 300 or $3\%$ of non-zero genes, and the genes with at least 100 or $2\%$ non-zero cells. After preprocessing, all 26, 830 cells with 13,118 genes were selected. The preprocessing step removed very few genes and cells because the dataset obtained from the original paper was filtered before cell-type annotation.
IPF The IPF dataset was obtained from human lung tissues to study the mechanisms and mediators driving fibrotic remodeling in lungs with pulmonary fibrosis [27]. The dataset contains 20 PF lungs and ten nonfibrotic controls. The preprocessing step excluded cells containing less than 1000 expressed genes and more than 25% mitochondrial genes. After preprocessing, 20,354 genes and 220,213 cells remained. Among all cells, 113,396 were clustered by Seurat v3 and annotated with canonical lineage-defining markers to 31 types. The authors reported no severe batch effects driven by the processing site or the sequencing batch, and thus no batch effect correction was applied in the annotation procedure.
COVID The COVID dataset was obtained from the *Fred Hutch Single-Cell Immunology Of SARS-CoV-2 Infection* data atlas [30]. We combined the top two largest datasets, SU [28] and STEPHENSON [29], to test CDI’s capability in handling a million-scale dataset. Both datasets were obtained from human peripheral blood mononuclear cells and preprocessed by excluding low-quality cells in the previous study [30]. SU contains 559,17 cells from 145 patients. STEPHENSON contains 691,683 cells from 120 patients. Both datasets were mapped to a reference PBMC dataset using Seurat v4 and annotated with SingleR [53]. The combined dataset COVID has five major populations: CD8 T, CD4 T, Mono, NK, B, other T, and others. Two layers of benchmark cell type labels further divide these cell populations into 31 and 57 cell types.

### Clustering methods

We applied six clustering methods to generate candidate label sets as the inputs of CDI. Three of them are specially designed for scRNA-seq clustering tasks (Seurat v3, CIDR, and SC3). The others are general clustering approaches. More details are in Table 3.

**Table 3:** Clustering method summary

Clustering method	Description	Reference
Hierarchical clustering	Bottom-up procedure to merge closest cells; complete linkage	[3]
K-means	Identify k-centers minimizing the within-cluster sum of squares	[2, 3]
Spectral clustering	Graph-based algorithm: eigenvectors of affinity matrix	[54]
CIDR	Dropout identification$+$PCA$+$hierarchical clustering	[5]
SC3	Consensus clustering (distance and correlations++PCA and Laplacian++K-means)	[10]
Seurat v3	PCA$+$Graph-based algorithm	[8]

For all datasets, UMI count matrices were first filtered as described in 5.4 before generating candidate label sets. We generated labels of the same range of cluster numbers for all methods for comparison. Generic clustering methods (hierarchical clustering, K-means, and spectral clustering) are not designed for large datasets like scRNA-seq. To make the clustering task feasible and efficient for these three generic methods, we input 200 PCs of scaled UMI counts rather than the raw UMI counts to these algorithms.

#### Hierarchical clustering

Function hclust() in R package stats [55] was applied with the complete agglomeration method.

#### KMeans

Function kmeans() in R package stats was applied with nstart = 3.

#### Spectral clustering

Function specc() in R package kernlab [56] was applied.

#### CIDR

We used default procedures of R pakcage cidr [57] for determining dropout candidates and calculating dissimilarity matrix. The nPC parameter in function scCluster() was set to be 200.

#### SC3

We used default procedures of R package SC3. For sc3() function, we set kmeans_nstart = 3, and svm_num_cells = 5000.

#### Seurat

We used default procedures of R package Seurat. UMI count matrix was first normalized with NormalizeData(). Method “vst” was applied to select 20% (SD1-SD4, T-CELL, and CORTEX) or 2,000 (RETINA and IPF) feature genes. Then after scaling the data, 20 principal components were used to feed the FindNeighbors function. Unlike other clustering methods we used, Seurat requires the resolution rather than the number of clusters as the tuning parameter. To compare with other methods under the same number of clusters, we searched a range of resolution values for Seurat to find one value corresponding to the desired number of clusters. For example, when we generated 10-cluster candidate label sets for SD1, we tried resolutions ranging from 0.1 to 6 and found that “resolution = 3” generated a 10-cluster label set. For each number of clusters, such resolution values are not unique, and we stopped at the first possible resolution during the search.

The RETINA dataset was generated from two experimental batches. We first applied Seurat Integration with the default setting to correct the batch effect. Then, the batch effect corrected dataset was used as the input to generate candidate label sets. Other procedures are the same as described above. The COVID dataset provided sparse PCA cell embeddings after batch effect correction under the “ref.spca” slot. We directly applied those cell embeddings as the input to generate candidate label sets from Seurat.

### Spearman correlation calculation

The Spearman correlation is the rank-based correlation between two vectors of the same length. It ranges from − 1 to 1, and a value of 1 occurs when two vectors have a perfect agreement in ranking.

We used the Spearman correlation to measure the ranking agreement between internal and external index scores for all datasets. Specifically, we applied different clustering methods with a wide range of tuning parameters to generate *L* candidate clustering label sets. Then, we calculated the external index scores between all the candidate label sets and the benchmark label set *b*, denoted by $\alpha _{\textrm{Ex},b}$. Each element of $\alpha _{\textrm{Ex},b}$ represents the external index score of a specific candidate label set compared with the benchmark label set *b*. On the other hand, we calculated the internal index scores of all the candidate label sets, denoted by $\alpha _{\textrm{In},b}$. Each element of $\alpha _{\textrm{In},b}$ represents the internal index score of a specific candidate label set. Since CDI, Connectivity, DB, SD-Scat, and XB mark better label sets with smaller values, we will replace $\alpha _{\textrm{In},b}$ by its negative.

The length $\alpha _{\textrm{Ex},b}$ and $\alpha _{\textrm{In},b}$ are the same, both equal to *L*. We then calculate the Spearman correlation between $\alpha _{\textrm{Ex},b}$ and $\alpha _{\textrm{In},b}$,

$$\begin{aligned} r_{b} = \frac{\texttt {Cov}\{R(\alpha _{\textrm{Ex},b}), R(\alpha _{\textrm{In},b})\}}{ [ \texttt {Var}\{R(\alpha _{\textrm{Ex},b})\} \texttt {Var}\{R(\alpha _{\textrm{In},b})\} ]^{1/2} }. \end{aligned}$$

Here, *R* transfers a vector into its ranking vector. Thus, the Spearman correlation can be viewed as the Pearson correlation between to ranking vectors.

To calculate the Spearman correlation for each pair of internal and external indices for all datasets with benchmark label set *b*, $b\in [B]$. Some datasets have two benchmark label sets (main-type and subtype), and thus two Spearman correlations were calculated.

## Supplementary Information

Additional file 1. Contains supplementary figures S1-S17, table S1, and notes 1-2.

Additional file 2. Review history.

## Data Availability

The murine colorectal carcinoma cell line CT26.WT dataset are available at the Genome Sequencing Archive (GSA): the accession is CRA008966 for the scRNA-seq data [58] and CRA008565 for the bulk RNA-seq data [59]. Five other public scRNA-seq datasets were also used in this study: T-CELL [60], CORTEX [61], RETINA [62], IPF [63], and COVID [64]. We used the CDI R packager version 0.99.12 in this study [65]. This R package has been submitted to the Bioconductor project. The development version of CDI is available from [66]. The scripts for to reproduce figures of the manuscript using this package are available at https://github.com/jfanglovestats/CDI_figures [66]. The package and source codes are licensed under GPL-3.

## References

[1] Shapiro E, Biezuner T, Linnarsson S (2013). Single-cell sequencing-based technologies will revolutionize whole-organism science. Nat Rev Genet..

[2] Lloyd S (1982). Least squares quantization in PCM. IEEE Trans Inf Theory..

[3] Hastie T, Tibshirani R, Friedman J. The elements of statistical learning. springer series in statistics. New York: Springer New York; 2001.

[4] Grün D, Lyubimova A, Kester L, Wiebrands K, Basak O, Sasaki N, Clevers H, Van Oudenaarden A. Single-cell messenger RNA sequencing reveals rare intestinal cell types. Nature. 2015;525(7568):251–5. 10.1038/nature14966 26287467

[5] Lin P, Troup M, Ho JW (2017). CIDR: Ultrafast and accurate clustering through imputation for single-cell RNA-seq data. Genome Biol..

[6] Wang B, Zhu J, Pierson E, Ramazzotti D, Batzoglou S (2017). Visualization and analysis of single-cell RNA-seq data by kernel-based similarity learning. Nat Methods..

[7] Wolf FA, Angerer P, Theis FJ (2018). SCANPY: large-scale single-cell gene expression data analysis. Genome Biol..

[8] Stuart T, Butler A, Hoffman P, Hafemeister C, Papalexi E, Mauck WM III, Hao Y, Stoeckius M, Smibert P, Satija R. Comprehensive integration of single-cell data. Cell. 2019;177(7):1888–902. 10.1016/j.cell.2019.05.031 PMC6687398 31178118

[9] Yang Y, Huh R, Culpepper HW, Lin Y, Love MI, Li Y (2019). SAFE-clustering: Single-cell aggregated (from ensemble) clustering for single-cell RNA-seq data. Bioinformatics..

[10] Kiselev VY, Kirschner K, Schaub MT, Andrews T, Yiu A, Chandra T, Natarajan KN, Reik W, Barahona M, Green AR, et al. SC3: consensus clustering of single-cell RNA-seq data. Nat Methods. 2017;14(5):483–6. 10.1038/nmeth.4236 PMC5410170 28346451

[11] Hubert L, Arabie P (1985). Comparing partitions. J Classif..

[12] Fowlkes EB, Mallows CL (1983). A method for comparing two hierarchical clusterings. J Am Stat Assoc..

[13] Vinh NX, Epps J, Bailey J (2010). Information theoretic measures for clusterings comparison: Variants, properties, normalization and correction for chance. J Mach Learn Res..

[14] Caliński T, Harabasz J (1974). A dendrite method for cluster analysis. Commun Stat-Theory Methods..

[15] Handl J, Knowles J. Exploiting the trade-off—the benefits of multiple objectives in data clustering. In: International conference on evolutionary multi-criterion optimization. Springer; 2005. p. 547–560.

[16] Davies DL, Bouldin DW (1979). A cluster separation measure. IEEE Trans Pattern Anal Mach Intell..

[17] Dunn JC (1974). Well-separated clusters and optimal fuzzy partitions. J Cybern..

[18] Baker FB, Hubert LJ (1975). Measuring the power of hierarchical cluster analysis. J Am Stat Assoc..

[19] Halkidi M, Batistakis Y, Vazirgiannis M (2001). On clustering validation techniques. J Intell Inf Syst..

[20] Xie XL, Beni G. A validity measure for fuzzy clustering. IEEE Trans Pattern Anal Mach Intell. 1991;13(8):841–7.

[21] Liu T, Yu H, Blair RH. Stability estimation for unsupervised clustering: A review. Wiley Interdiscip Rev Comput Stat. 2022;14(6):e1575. 10.1002/wics.1575 PMC9787023 36583207

[22] Klein AM, Mazutis L, Akartuna I, Tallapragada N, Veres A, Li V, Peshkin L, Weitz DA, Kirschner MW. Droplet barcoding for single-cell transcriptomics applied to embryonic stem cells. Cell. 2015;161(5):1187–201. 10.1016/j.cell.2015.04.044 PMC4441768 26000487

[23] Zheng GXY, Terry JM, Belgrader P, Ryvkin P, Bent ZW, Wilson R, Ziraldo, S.B., Wheeler, TD, McDermott GP, Zhu J, Gregory MT, Shuga J, Montesclaros L, Underwood JG, Masquelier DA, Nishimura SY, Schnall-Levin M, Wyatt PW, Hindson CM, Bharadwaj R, Wong A, Ness KD, Beppu LW, Deeg HJ, McFarland C, Loeb KR, Valente WJ, Ericson NG, Stevens EA, Radich JP, Mikkelsen TS, Hindson BJ, Bielas JH. Massively parallel digital transcriptional profiling of single cells. Nat Commun. 2017;8:14049. 10.1038/ncomms14049. 10.1038/ncomms14049 PMC5241818 28091601

[24] Christian LS, Wang L, Lim B, Deng D, Wu H, Wang XF, Li QJ. Resident memory T cells in tumor-distant tissues fortify against metastasis formation. Cell Rep. 2021;35(6). 10.1016/j.celrep.2021.109118. 10.1016/j.celrep.2021.109118 PMC8204287 33979626

[25] Hrvatin S, Hochbaum DR, Nagy MA, Cicconet M, Robertson K, Cheadle L, Zilionis R, Ratner A, Borges-Monroy R, Klein AM, et al. Single-cell analysis of experience-dependent transcriptomic states in the mouse visual cortex. Nat Neurosci. 2018;21(1):120–9. 10.1038/s41593-017-0029-5 PMC5742025 29230054

[26] Shekhar K, Lapan SW, Whitney IE, Tran NM, Macosko EZ, Kowalczyk M, Adiconis X, Levin JZ, Nemesh J, Goldman M, et al. Comprehensive classification of retinal bipolar neurons by single-cell transcriptomics. Cell. 2016;166(5):1308–23. 10.1016/j.cell.2016.07.054 PMC5003425 27565351

[27] Habermann AC, Gutierrez AJ, Bui LT, Yahn SL, Winters NI, Calvi CL, Peter L, Chung MI, Taylor CJ, Jetter C, et al. Single-cell RNA sequencing reveals profibrotic roles of distinct epithelial and mesenchymal lineages in pulmonary fibrosis. Sci Adv. 2020;6(28):eaba1972. 10.1126/sciadv.aba1972 PMC7439444 32832598

[28] Su Y, Chen D, Lausted C, Yuan D, Choi J, Dai C, Voillet V, Scherler K, Troisch P, Duvvuri V, et al. Multiomic immunophenotyping of COVID-19 patients reveals early infection trajectories. bioRxiv. 2020: 2020.07.27.224063.

[29] Stephenson E, Reynolds G, Botting RA, Calero-Nieto FJ, Morgan MD, Tuong ZK, Bach K, Sungnak W, Worlock KB, Yoshida M, Kumasaka N. Single-cell multi-omics analysis of the immune response in COVID-19. Nat Med. 2021;27(5):904–16. 10.1038/s41591-021-01329-2 PMC8121667 33879890

[30] Tian Y, Carpp LN, Miller HE, Zager M, Newell EW, Gottardo R. Single-cell immunology of SARS-CoV-2 infection. Nat Biotechnol. 2022;40(1):30–41. 10.1038/s41587-021-01131-y PMC9414121 34931002

[31] Chernoff H, Lehmann EL. The use of maximum likelihood estimates in $$\chi ^2$$ tests for goodness of fit. Ann Math Statist. 1954;25(3):579–86. 10.1214/aoms/1177728726.

[32] Townes FW, Hicks SC, Aryee MJ, Irizarry RA (2019). Feature selection and dimension reduction for single-cell RNA-Seq based on a multinomial model. Genome Biol..

[33] Svensson V (2020). Droplet scRNA-seq is not zero-inflated. Nat Biotechnol..

[34] Kim TH, Zhou X, Chen M. Demystifying “drop-outs” in single-cell UMI data. Genome Biol. 2020;21(1):196. 10.1186/s13059-020-02096-y. 10.1186/s13059-020-02096-y PMC7412673 32762710

[35] Cao Y, Kitanovski S, Küppers R, Hoffmann D (2021). UMI or not UMI, that is the question for scRNA-seq zero-inflation. Nat Biotechnol..

[36] Brennecke P, Anders S, Kim JK, Kołodziejczyk AA, Zhang X, Proserpio V, Baying B, Benes V, Teichmann SA, Marioni JC, et al. Accounting for technical noise in single-cell RNA-seq experiments. Nat Methods. 2013;10(11):1093. 10.1038/nmeth.2645 24056876

[37] Stuart T, Butler A, Hoffman P, Hafemeister C, Papalexi E, Mauck WM 3rd, Hao Y, Stoeckius M, Smibert P, Satija R. Comprehensive integration of single-cell data. Cell. 2019;177(7):1888–190221. 10.1016/j.cell.2019.05.031. 10.1016/j.cell.2019.05.031 PMC6687398 31178118

[38] Hao Y, Hao S, Andersen-Nissen E, Mauck WM, Zheng S, Butler A, Lee MJ, Wilk AJ, Darby C, Zager M, Hoffman P, Stoeckius M, Papalexi E, Mimitou EP, Jain J, Srivastava A, Stuart T, Fleming LM, Yeung B, Rogers AJ, McElrath JM, Blish CA, Gottardo R, Smibert P, Satija R (2021). Integrated analysis of multimodal single-cell data. Cell..

[39] Becht E, McInnes L, Healy J, Dutertre CA, Kwok IWH, Ng LG, Ginhoux F, Newell EW. Dimensionality reduction for visualizing single-cell data using UMAP. Nat Biotechnol. 2018. 10.1038/nbt.4314. 10.1038/nbt.4314 30531897

[40] Zappia L, Phipson B, Oshlack A (2017). Splatter: simulation of single-cell RNA sequencing data. Genome Biol..

[41] Rousseeuw PJ (1987). Silhouettes: a graphical aid to the interpretation and validation of cluster analysis. J Comput Appl Math..

[42] Peyvandipour A, Shafi A, Saberian N, Draghici S (2020). Identification of cell types from single cell data using stable clustering. Sci Rep..

[43] Liu B, Li C, Li Z, Wang D, Ren X, Zhang Z (2020). An entropy-based metric for assessing the purity of single cell populations. Nat Commun..

[44] Jiang H, Sohn LL, Huang H, Chen L (2018). Single cell clustering based on cell-pair differentiability correlation and variance analysis. Bioinformatics..

[45] Spearman C. The proof and measurement of association between two things. 1961.

[46] Fang J, Chan C, Owzar K, Wang L, Qin D, Li QJ, Xie, J. CDI package: Genome Biology Publication. Zenodo; 2022. 10.5281/zenodo.7007246. Accessed 18 Aug 2022.

[47] Brock G, Pihur V, Datta S, Datta S (2008). clValid: An R Package for Cluster Validation. J Stat Softw..

[48] Desgraupes B. clusterCrit: clustering indices. 2018. R package version 1.2.8. https://CRAN.R-project.org/package=clusterCrit. Accessed 18 Aug 2022.

[49] Jiang L, Schlesinger F, Davis CA, Zhang Y, Li R, Salit M, Gingeras TR, Oliver B (2011). Synthetic spike-in standards for RNA-seq experiments. Genome Res..

[50] Huang M, Wang J, Torre E, Dueck H, Shaffer S, Bonasio R, Murray JI, Raj A, Li M, Zhang NR (2018). SAVER: gene expression recovery for single-cell RNA sequencing. Nat Methods..

[51] Rodriguez A, Laio A (2014). Clustering by fast search and find of density peaks. Science..

[52] Blondel VD, Guillaume JL, Lambiotte R, Lefebvre E (2008). Fast unfolding of communities in large networks. J Stat Mech: Theory Exp..

[53] Aran D, Looney AP, Liu L, Wu E, Fong V, Hsu A, Chak S, Naikawadi RP, Wolters PJ, Abate AR (2019). Reference-based analysis of lung single-cell sequencing reveals a transitional profibrotic macrophage. Nat Immunol..

[54] Ng A, Jordan M (2001). Weiss Y.

[55] Team RC, et al. R: A language and environment for statistical computing. 2013.

[56] Karatzoglou A, Smola A, Hornik K, Zeileis A (2004). kernlab-an S4 package for kernel methods in R. J Stat Softw..

[57] Lin P, Troup M. Cidr: Clustering through imputation and dimensionality reduction. 2020. R package version 0.1.5.

[58] Fang J, Chan C, Owzar K, Wang L, Qin D, Li QJ, Xie J. Clustering Deviation Index (CDI): a robust and accurate internal measure for evaluating scRNA-seq data clustering. GSA; 2022. ScRNA-seq Dataset. https://ngdc.cncb.ac.cn/gsa/browse/CRA008966. Accessed 1 Dec 2022. 10.1186/s13059-022-02825-5 PMC9793368 36575517

[59] Fang J, Chan C, Owzar K, Wang L, Qin D, Li QJ, Xie J. Clustering Deviation Index (CDI): a robust and accurate internal measure for evaluating scRNA-seq data clustering. GSA; 2022. RNA-seq Dataset. https://ngdc.cncb.ac.cn/gsa/browse/CRA008565. Accessed 18 Aug 2022. 10.1186/s13059-022-02825-5 PMC9793368 36575517

[60] Christian LS, Wang L, Lim B, Deng D, Wu H, Wang XF, Li QJ. Resident memory T cells in tumor-distant tissues fortify against metastasis formation. Mendeley Data; 2022. Datasets. https://data.mendeley.com/datasets/3f4rsk96kf/4. Accessed 30 Oct 2022.

[61] Hrvatin S, Hochbaum DR, Nagy MA, Cicconet M, Robertson K, Cheadle L, Zilionis R, Ratner A, Borges-Monroy R, Klein AM, et al. Single-cell analysis of experience-dependent transcriptomic states in the mouse visual cortex. Gene Expression Omnibus; 2017. Datasets. https://www.ncbi.nlm.nih.gov/geo/query/acc.cgi?acc=GSE102827. Accessed 18 Aug 2022.

[62] Shekhar K, Lapan SW, Whitney IE, Tran NM, Macosko EZ, Kowalczyk M, Adiconis X, Levin JZ, Nemesh J, Goldman M, et al. Comprehensive classification of retinal bipolar neurons by single-cell transcriptomics. Gene Expression Omnibus; 2016. Datasets. https://www.ncbi.nlm.nih.gov/geo/query/acc.cgi?acc=GSE81905. Accessed 18 Aug 2022. 10.1016/j.cell.2016.07.054 PMC5003425 27565351

[63] Habermann AC, Gutierrez AJ, Bui LT, Yahn SL, Winters NI, Calvi CL, Peter L, Chung MI, Taylor CJ, Jetter C, et al. Single-cell RNA sequencing reveals profibrotic roles of distinct epithelial and mesenchymal lineages in pulmonary fibrosis. Gene Expression Omnibus; 2019. Datasets. https://www.ncbi.nlm.nih.gov/geo/query/acc.cgi?acc=GSE135893. Accessed 18 Aug 2022. 10.1126/sciadv.aba1972 PMC7439444 32832598

[64] Tian Y, Carpp LN, Miller HE, Zager M, Newell EW, Gottardo R. Single-cell immunology of SARS-CoV-2 infection. Fred Hutch; 2022. Datasets. https://atlas.fredhutch.org/fredhutch/covid/. Accessed 18 Aug 2022. 10.1038/s41587-021-01131-y PMC9414121 34931002

[65] Fang J, Chan C, Owzar K, Wang L, Qin D, Li QJ, Xie J. Reproduce code: Genome Biology Publication. Zenodo; 2022. 10.5281/zenodo.7005019. Accessed 18 Aug 2022.

[66] Fang J, Chan C, Owzar K, Wang L, Qin D, Li QJ, Xie J. CDI: Clustering Deviation Index (CDI). GitHub; 2021. https://github.com/jichunxie/CDI. Accessed 18 Aug 2022. 10.1186/s13059-022-02825-5 PMC9793368 36575517

