# Supplementary material for: Clustering Deviation Index (CDI): a robust and accurate internal measure for evaluating scRNA-seq data clustering
Source: Genome Biol. 2022 Dec 27;23:269. doi: 10.1186/s13059-022-02825-5 (PMC9793368; doi:10.1186/s13059-022-02825-5)
Supplement: Supplementary file 1 — Additional file 1. Contains supplementary figures S1-S17, table S1, and notes 1-2. [file 13059_2022_2825_MOESM1_ESM.pdf]

1 Supplementary Figures

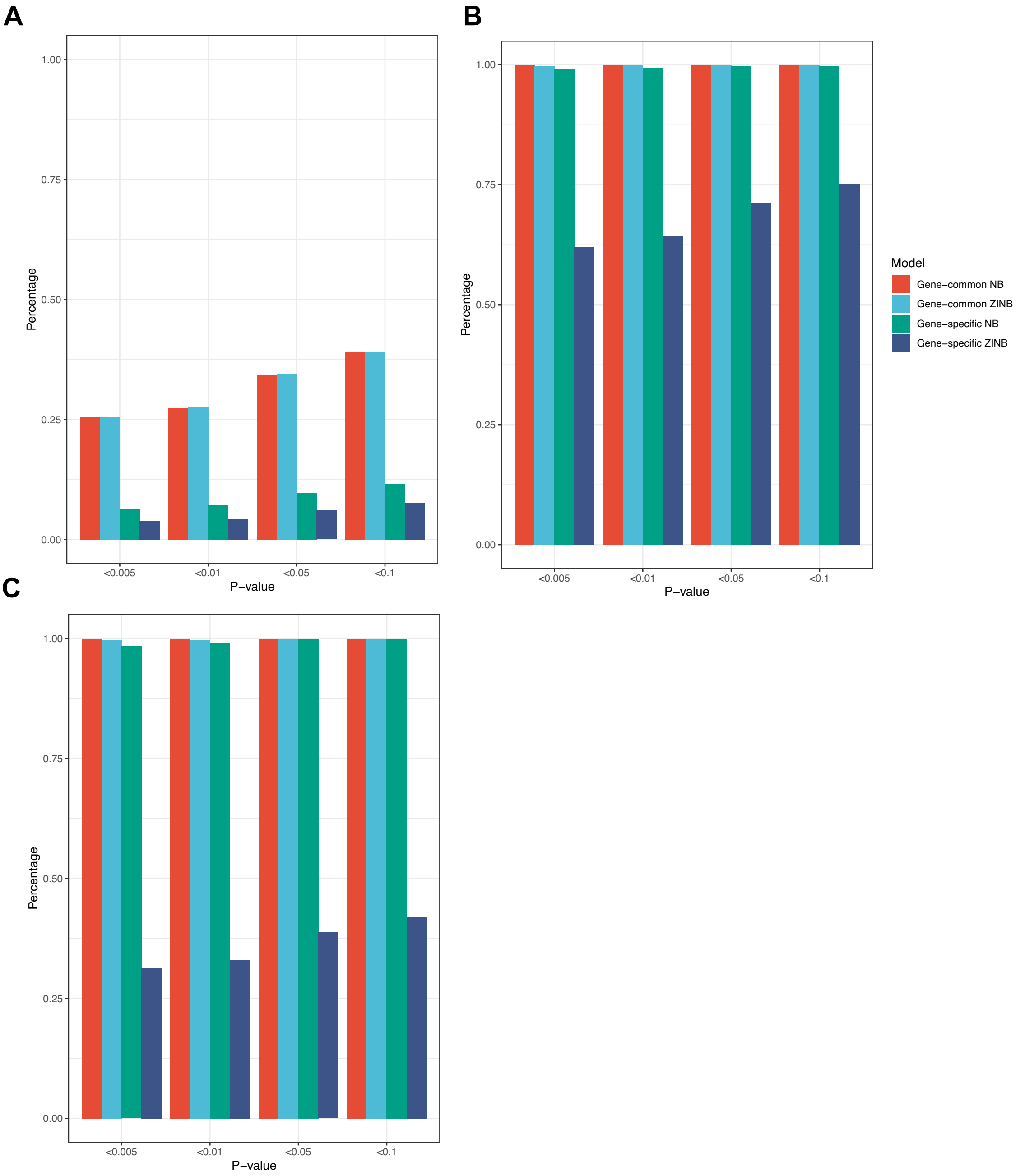

Fig. S1: Percentages of genes rejected by the Pearson "goodness-of-fit" tests in CT26.WT when the type I errors were set at 0.5%, 1%, 5%, and 10%. We checked the distributions of the raw UMI counts (A), the FPKM normalized counts (B), and the TPM normalized counts (C). The gene length for calculating FPKM and TPM was obtained from R package `EDASeq` by mapping the gene emsembl IDs to the external database.

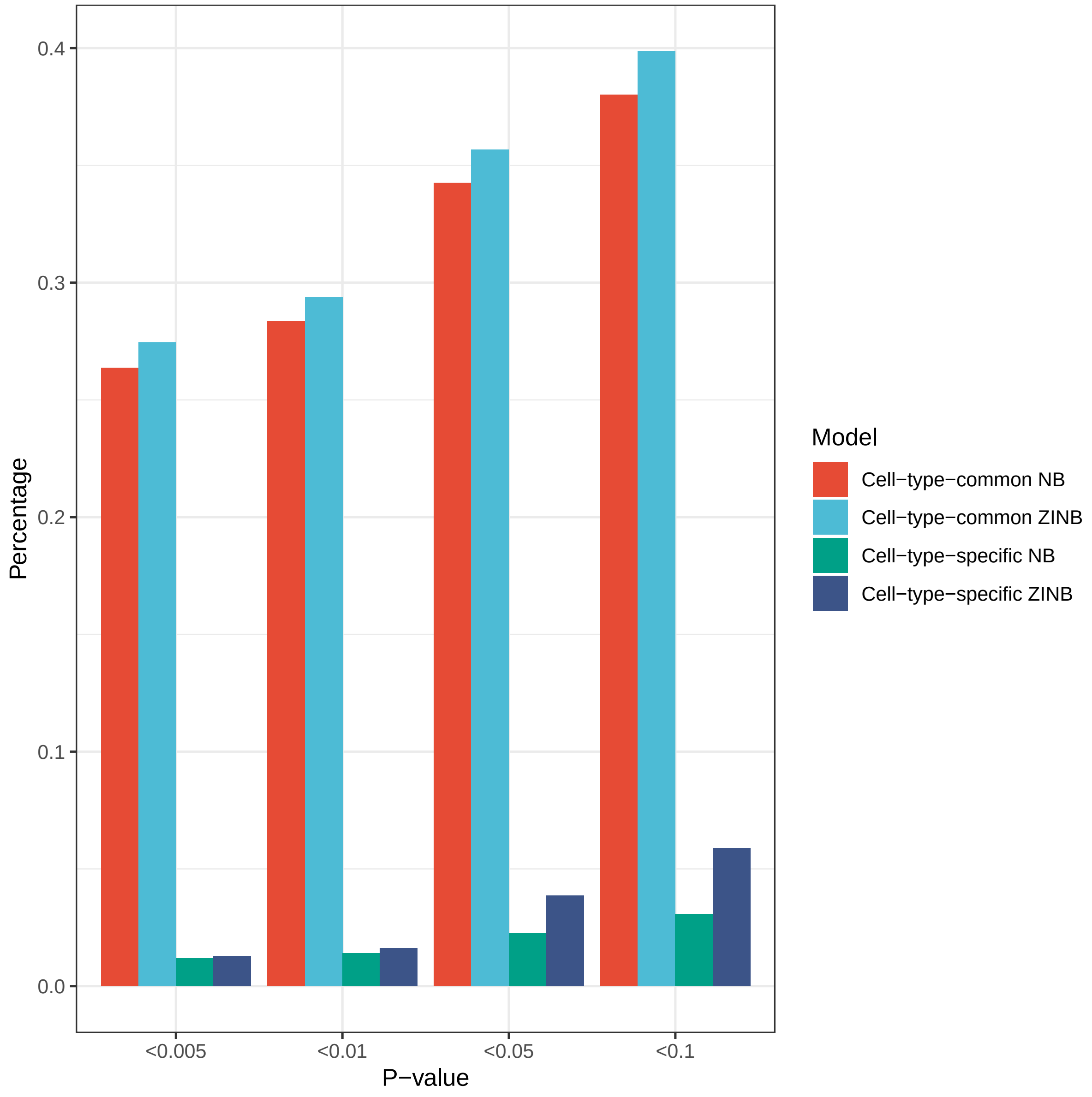

Fig. S2: Percentages of genes rejected by the cell-type-specific "goodness-of-fit" test in T-CELL when the type I errors were set at 0.5%, 1%, 5%, and 10%. The tests were performed on 7,893 genes.

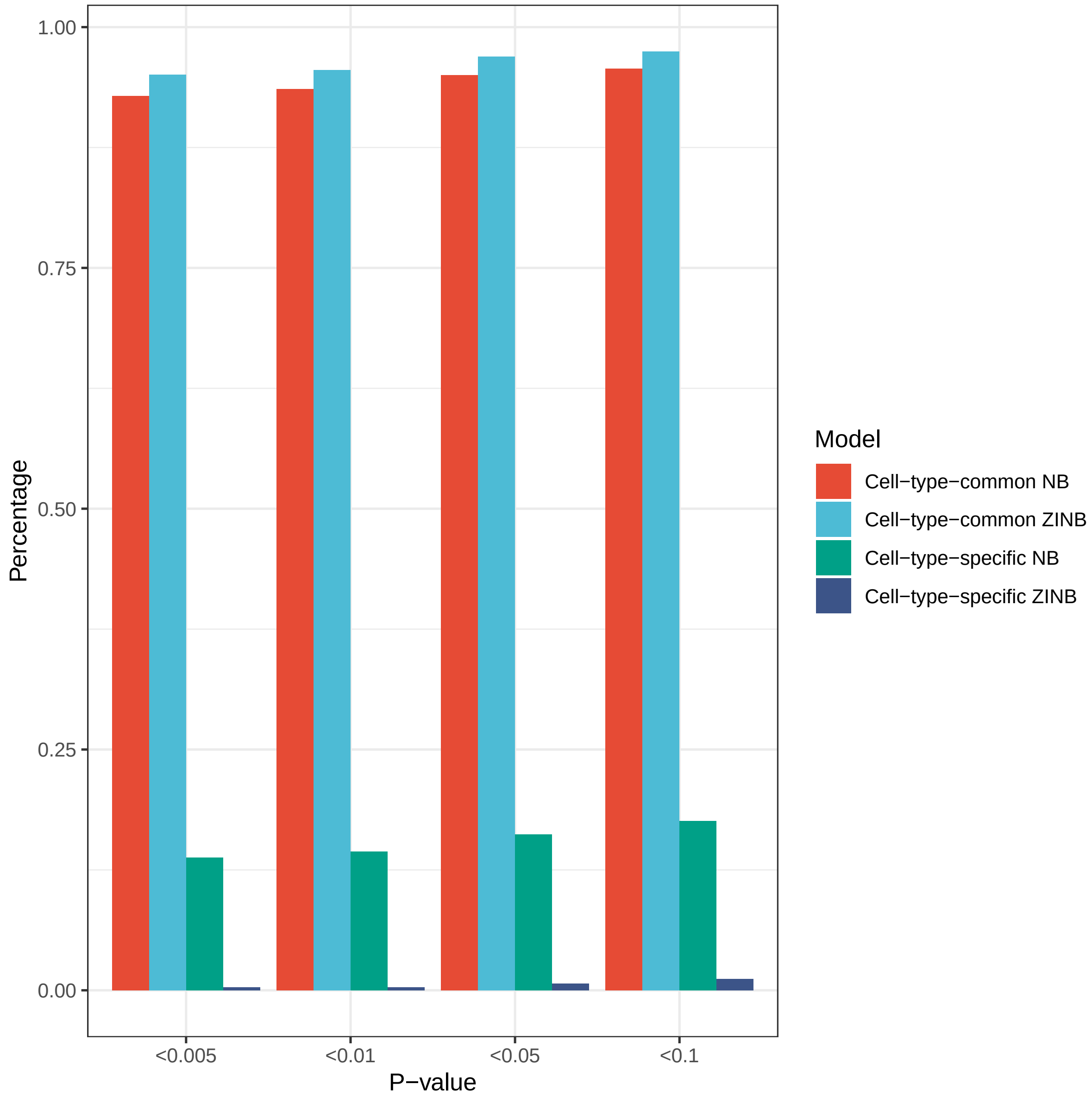

Fig. S3: Percentages of genes rejected by the cell-type-specific "goodness-of-fit" test in CORTEX when the type I errors were set at 0.5%, 1%, 5%, and 10%. The tests were performed on 12,887 genes.

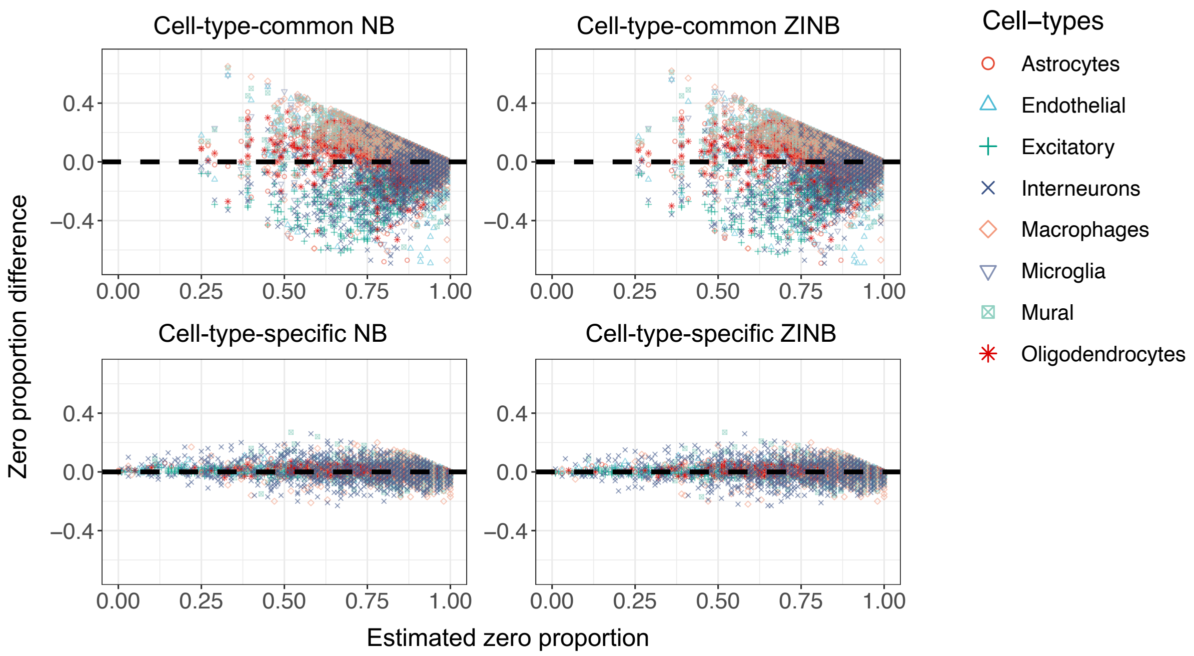

Fig. S4: Four scatter plots show the difference between the observed and estimated UMI count zero proportions versus the estimated UMI count zero proportions in CORTEX under the four families of distributions. Each dot represents a gene. The cell-type-common NB and ZINB families under-estimated the UMI zero proportions; the cell-type-specific NB and ZINB estimated well. To reduce the size of the plot, $2,000$ genes were randomly selected for visualization.

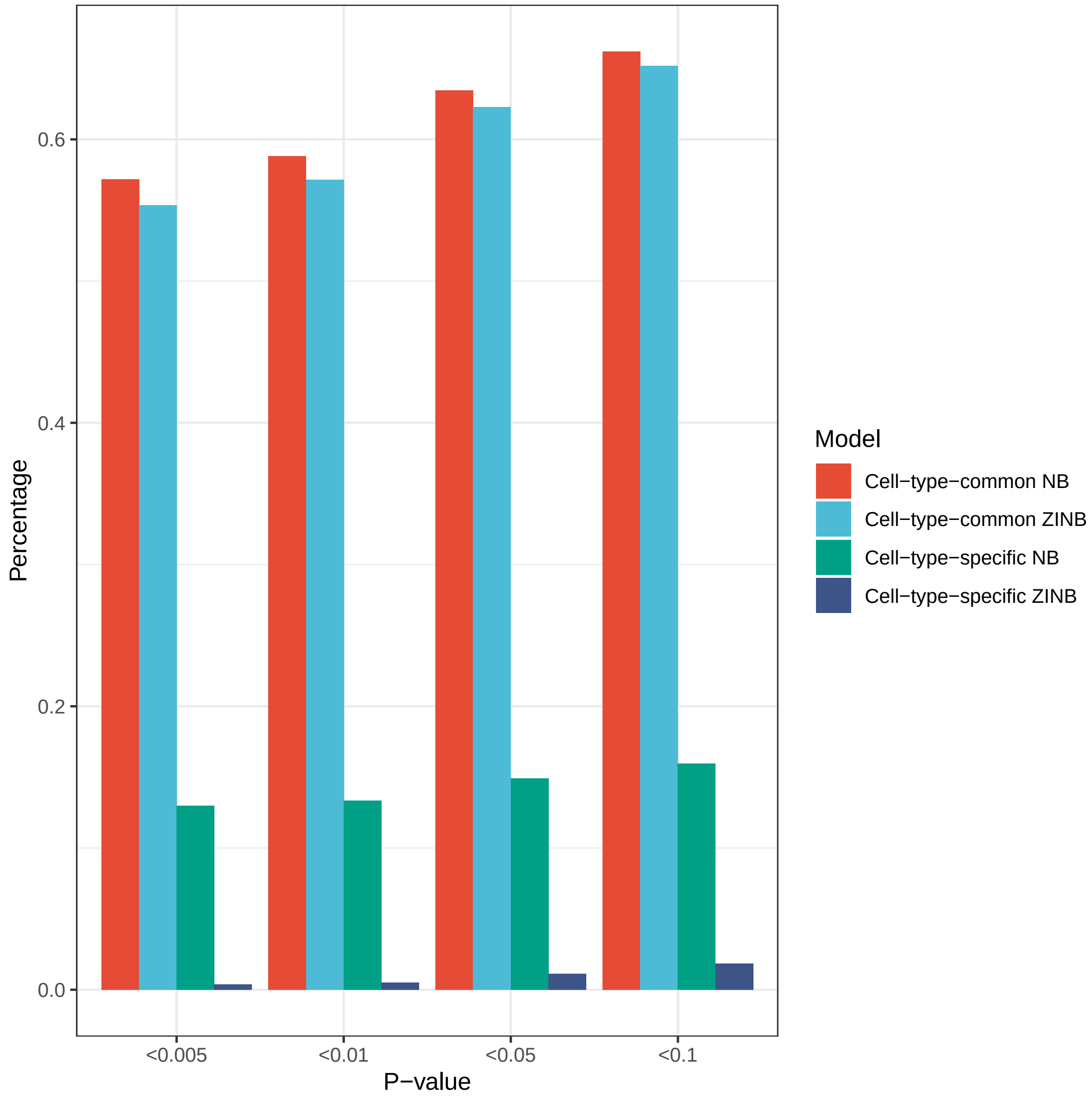

Fig. S5: Percentages of genes rejected by the cell-type-specific "goodness-of-fit" test in RETINA Batch 1 when the type I errors were set at 0.5%, 1%, 5%, and 10%. The tests were performed on 12,000 genes with 13,666 cells in Batch 1 to avoid the impact of the batch effect.

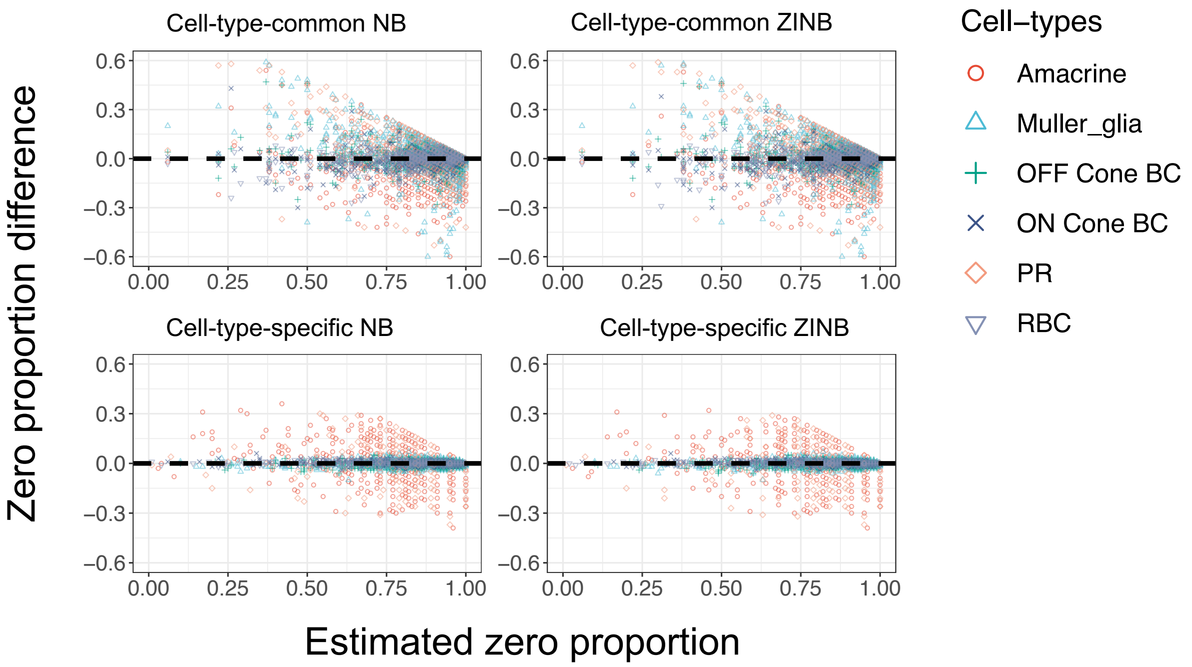

Fig. S6: Four scatter plots show the difference between the observed and estimated UMI count zero proportions versus the estimated UMI count zero proportions in RETINA Batch 1 under the four families of distributions. Each dot represents a gene. The cell-type-common NB and ZINB families under-estimated the UMI zero proportions; the cell-type-specific NB and ZINB estimated well. To reduce the size of the plot, $2,000$ genes were randomly selected for visualization. The PR and Amacrine cell-types have relatively small number of cells (PR, 0.278%, 38 cells; Amacrine, 0.329%, 45 cells), and thus the model fittings are less accurate, especially the models are fitted using only the training samples.

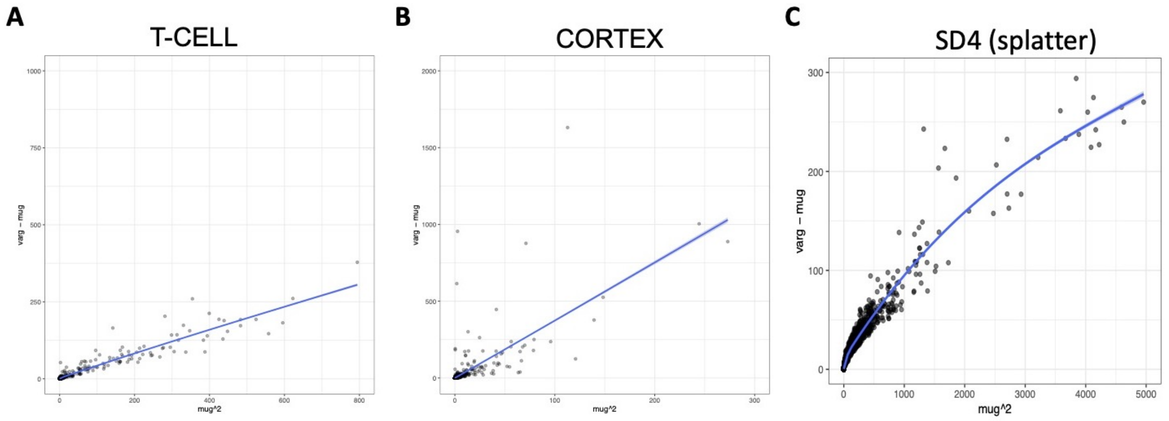

Fig. S7: The mean-variance trends of UMI counts in T-CELL (A), CORTEX (B), and SD4 (C). Each dot represents a gene. The x-axis is UMI mean square μ^2; the y-axis is the UMI variance minus the UMI mean, $\sigma^2 - \mu$. Each dot represents a gene. The smooth curve is fitted by the generalized additive model.

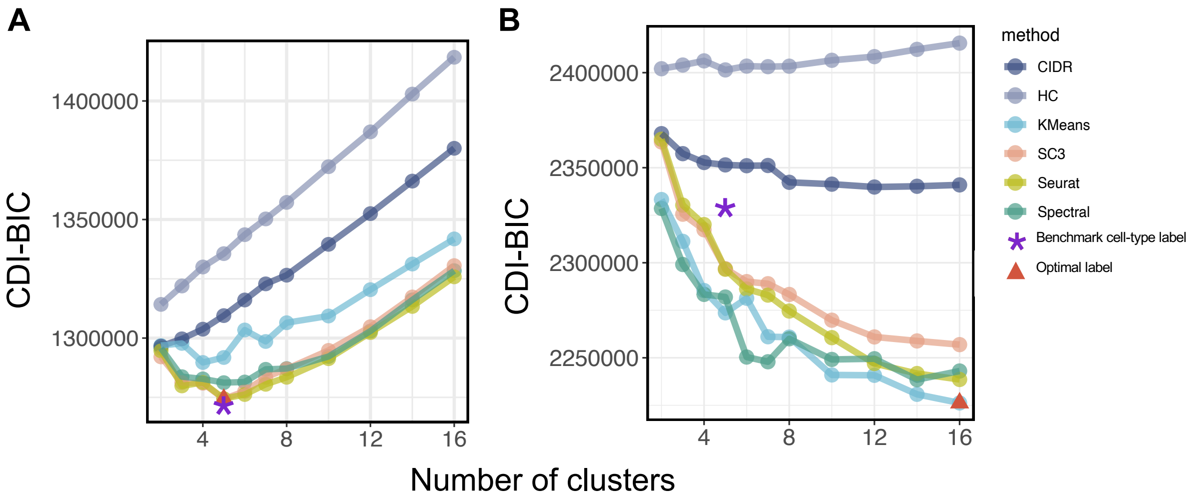

Fig. S8: WDS- and VST-selected feature genes lead to different CDI-BIC scores of the candidate label sets for T-CELL. (A) CDI scores based on the 500 WDS-selected feature genes; (B) CDI scores based on the 500 VST-selected feature genes. The x-axis labels the cluster number; the y-axis labels the CDI scores. Each dot represents a label set (either the benchmark or the candidate label set). The red triangle marks the CDI-selected label set; the purple star marks the benchmark label set.

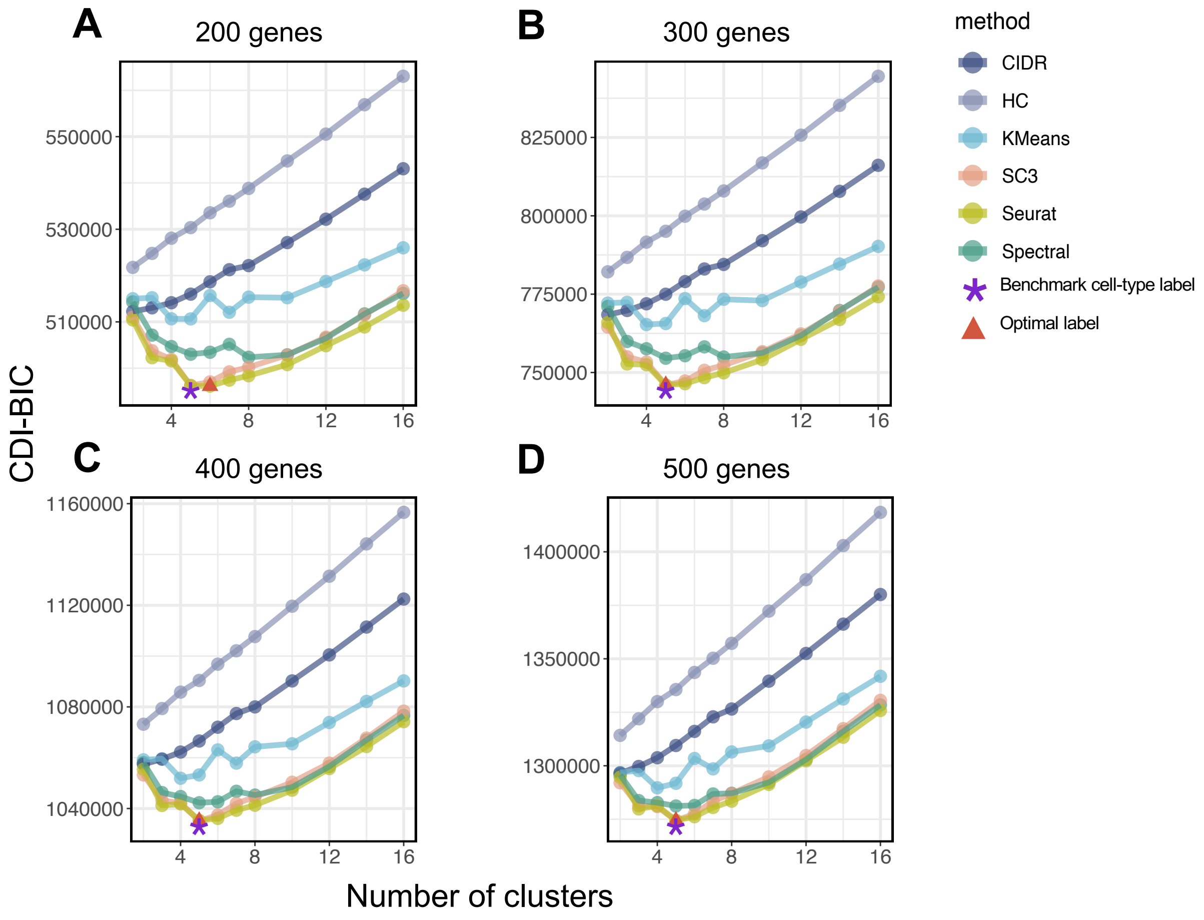

Fig. S9: CDI performance on T-CELL using different numbers of WDS-selected feature genes. (A) Using 200 feature genes, CDI selected the six-cluster label set generated by Seurat. (B) Using 300 feature genes, CDI selected the five-cluster label set generated by Seurat. (C) Using 400 feature genes, CDI selected the five-cluster label set generated by SC3. (D) Using 500 feature genes, CDI selected the five-cluster label set generated by SC3. In all plots, the x-axis labels the cluster numbers, the y-axis labels the CDI sores, and the colors represent different clustering methods. The red triangle marks the CDI-selected label set; the purple star marks the benchmark label set.

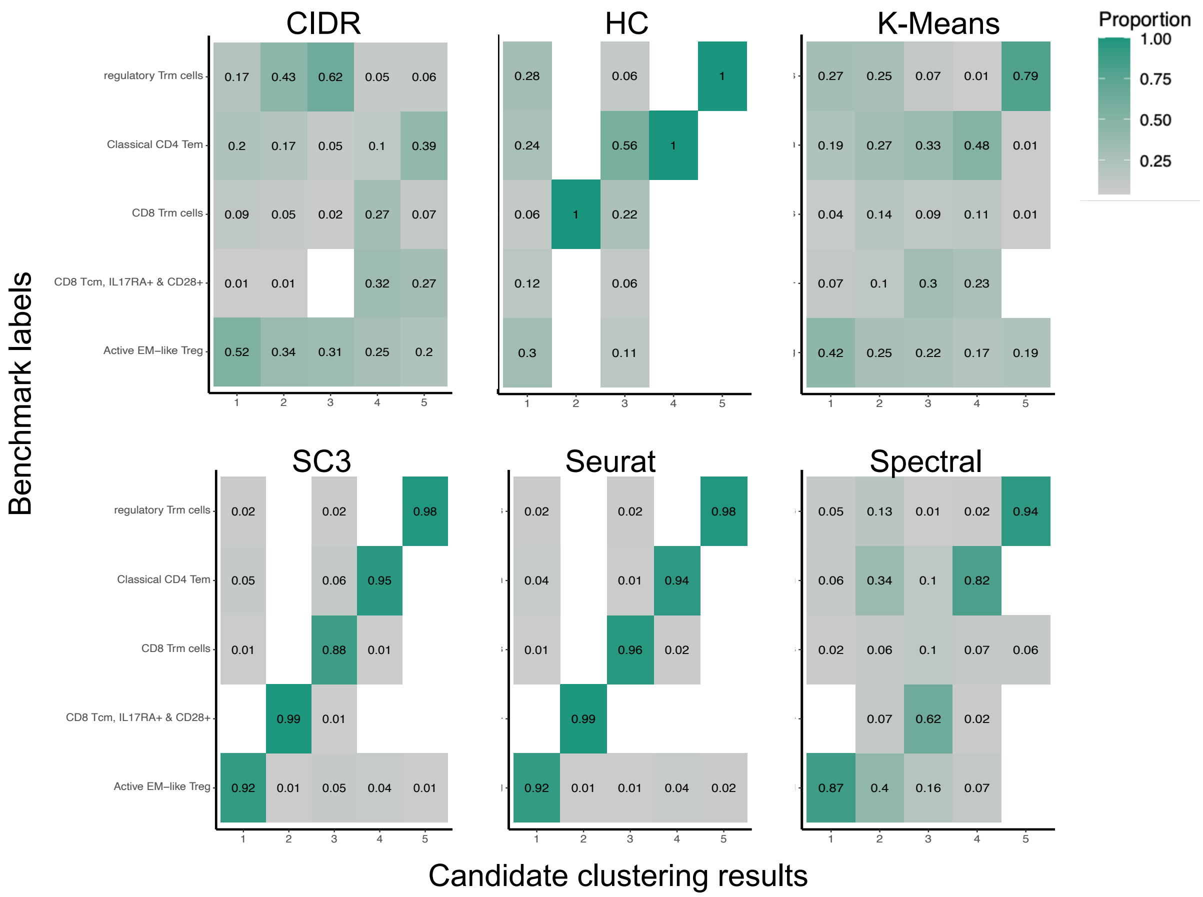

Fig. S10: The heatmaps show the cell proportions from the benchmark label sets for each cluster in the selected label sets for T-CELL. The x-axis labels the clusters in the five-cluster candidate label sets derived by various clustering methods, and the y-axis labels the benchmark cell types. The color of each rectangle represents the benchmark cell-type proportions in each selected label set cluster. Each column adds up to 1. For example, in SC3, 92% of the cluster 1 cells are active EM-like Treg cells.

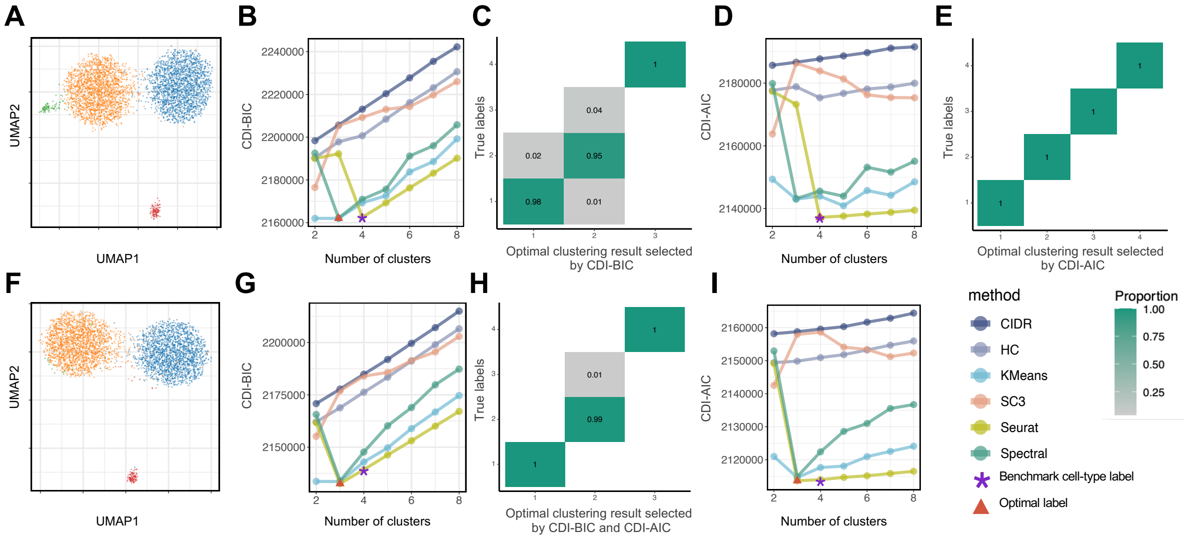

Fig. S11: Both CDI-AIC and CDI-BIC perform well on the cell populations with rare cell types. (A)-(E) show the results for the simulated dataset with two abundant cell types (each with $2,000$ cells) and two rare cell types, called RC1 (85 cells) and RC2 (100 cells). RC1 is more similar to the two abundant cell types. (F)-(I) show the results for the simualted dataset with with two abundant cell types (each with $2,000$ cells), RC1 with 20 cells, and RC2 with 100 cells. (A) and (F) show the UMAPs based on the WDS-selected feature genes; (B) and (G) show the CDI-BIC scores of the candidate and benchmark label sets; (D) and (I) show the CDI-AIC scores of the candidate and benchmark label sets. CDI-AIC and CDI-BIC selected different optimal label sets: (C), (E), and (H)are heatmaps of the cell proportions from the benchmark label sets for each cluster in the selected label sets. (C) shows the heatmap for CDI-BIC selected label set for the dataset with 85 cells in RC1; (E) shows the heatmap for CDI-AIC selected label set for the dataset with 85 cells in RC1; (H) shows the heatmap for the label set selected by both CDI-AIC and CDI-BIC for the dataset with 20 cells in RC1.

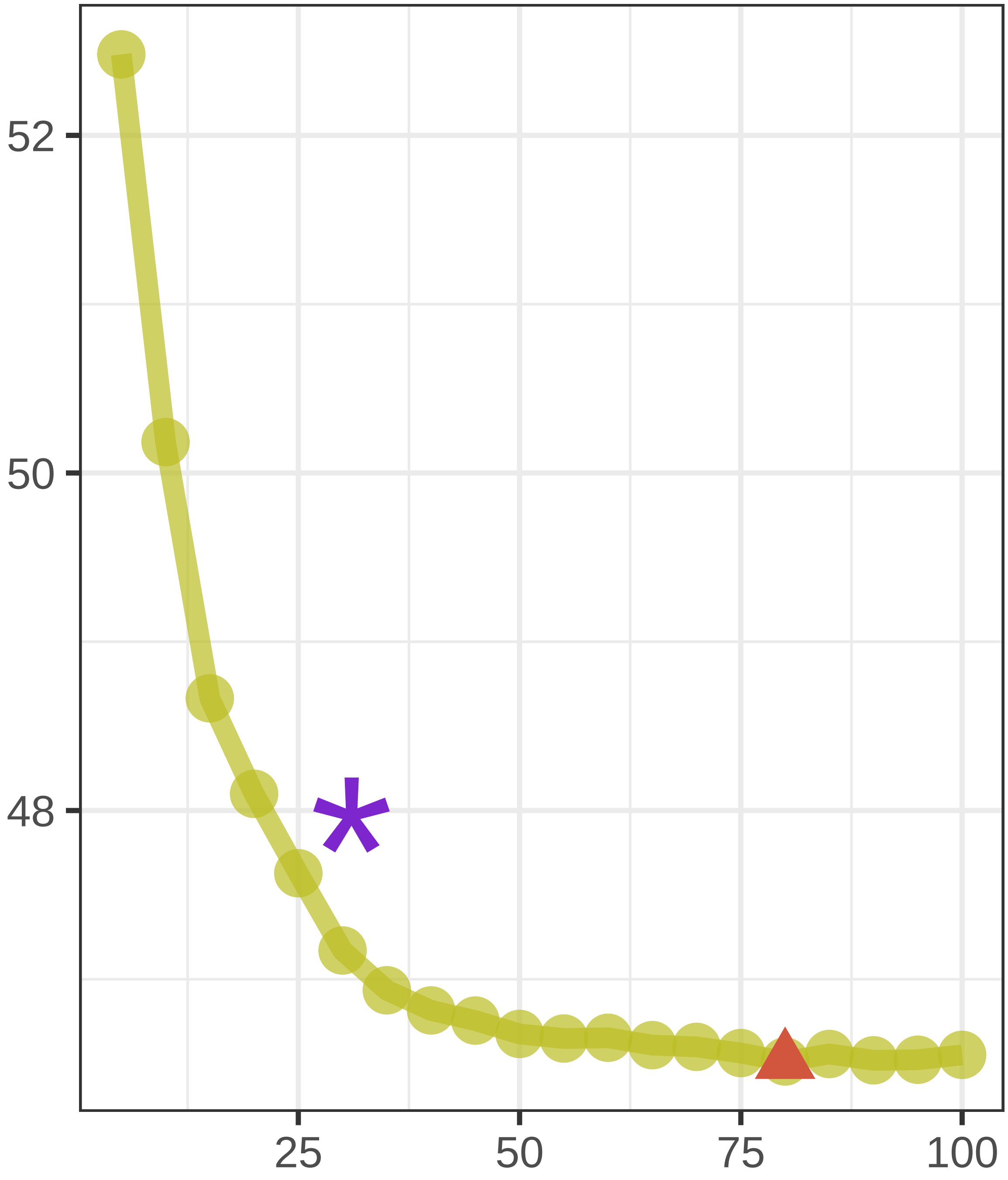

Fig. S12: The lineplot shows the CDI-BIC scores of 20 Seurat v3 generated candidate label sets for IPF. The triangle marks the CDI score of the CDI-selected label set; the star marks the CDI score of the benchmark label set.

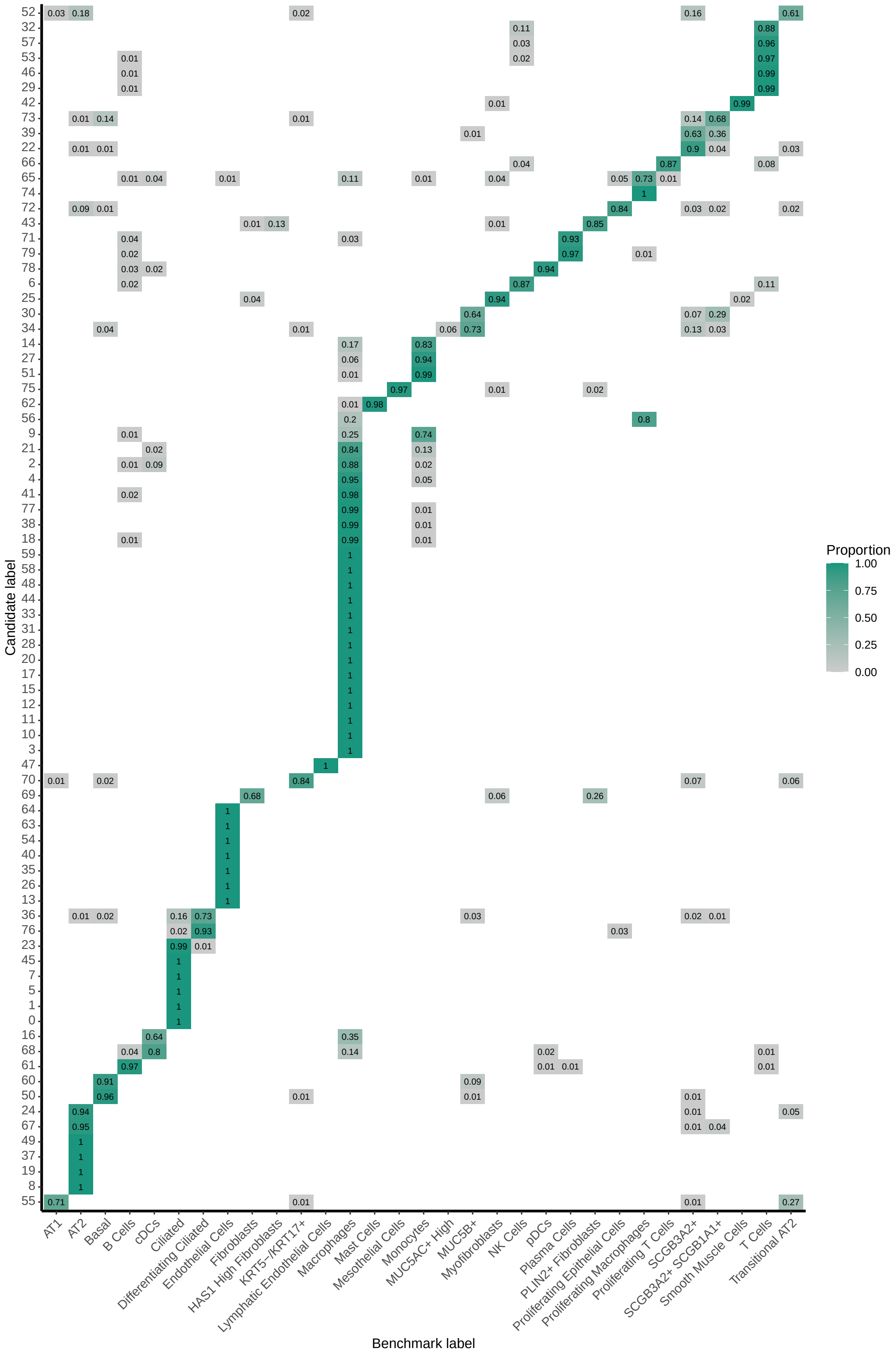

Fig. S13: The heatmap of the cell proportions from the benchmark label sets for each cluster in the CDI-BIC selected label set for IPF. Each row adds up to 1.

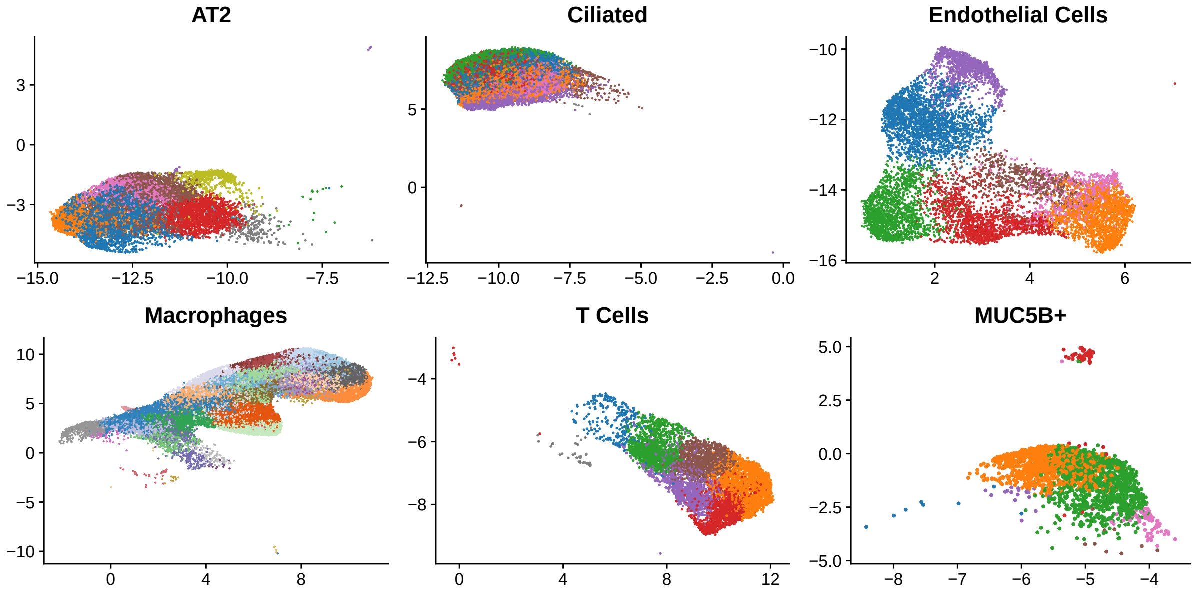

Fig. S14: The UMAPs for the AT2 (9, 311), ciliated (14, 671), endothelial (9, 243 cells), macrophages (38, 923), T (6, 703), and MUC5B+ (2, 433) cells in the IPF benchmark label set. The numbers in the bracket show the cell number in each cell type. The cells are colored based on the cell types in the CDI-BIC selected label set.

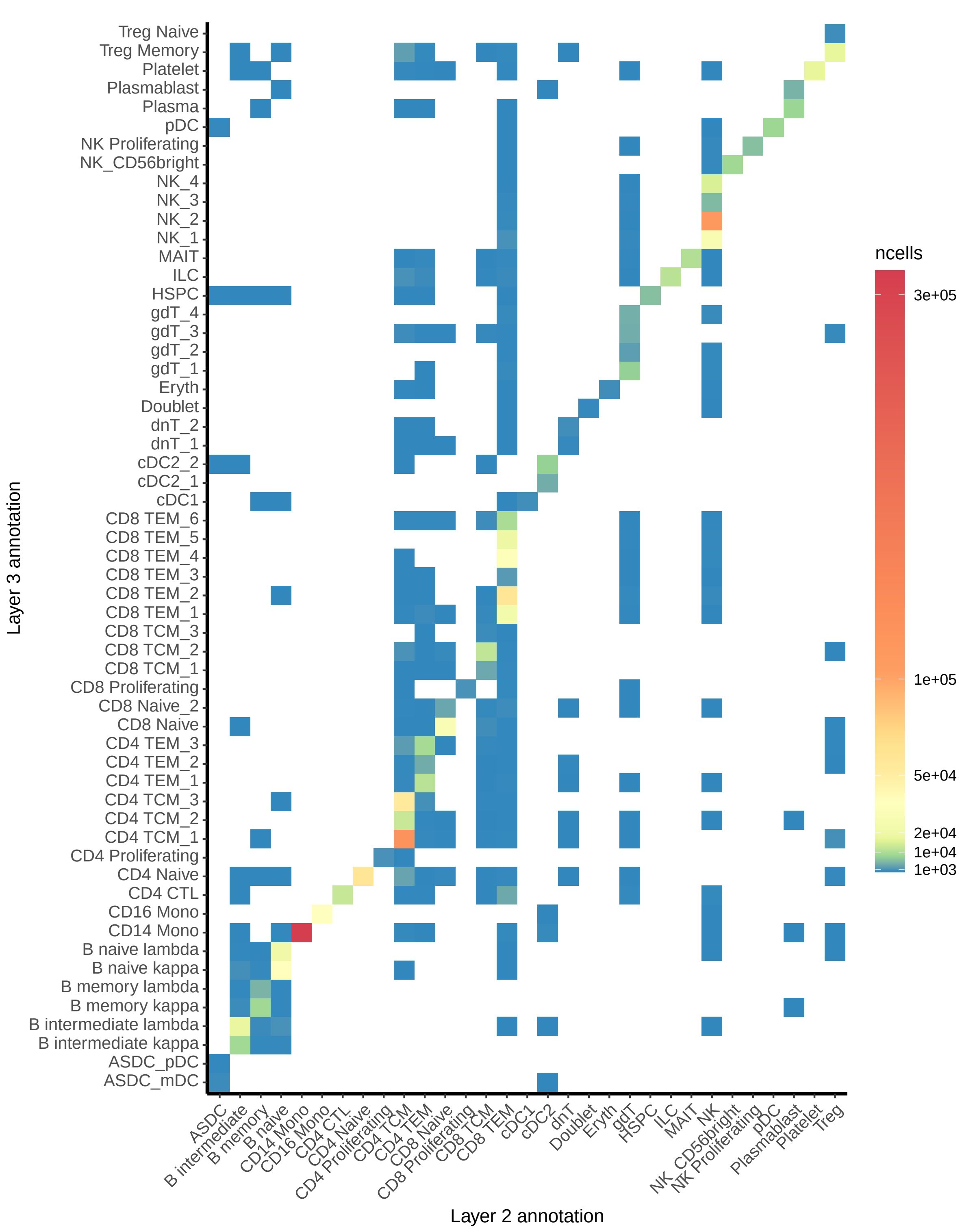

Fig. S15: The heatmap shows the number of cells from the layer 3 annotation for each cell type in the layer 2 annotation provided in the previous study of COVID dataset. The x-axis labels the layer 2 annotation, and the y-axis labels the layer 3 annotation. The color of each rectangle represents the number of cells.

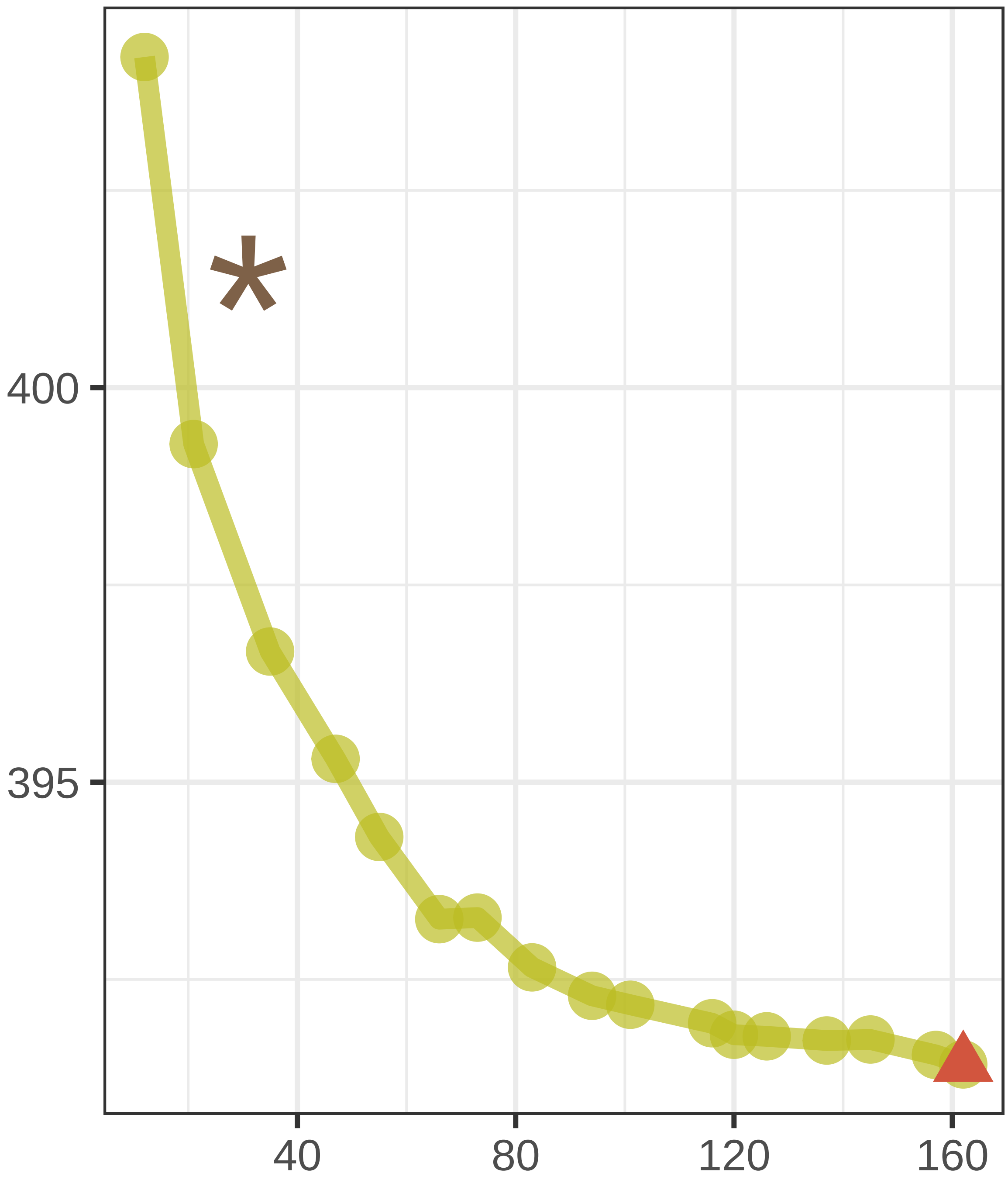

Fig. S16: CDI-BIC lineplot for the COVID dataset with candidate labels generating from Seurat v3. The number of clusters corresponds to Seurat resolutions 0.1, 0.5, 1.0, 1.5, 2.0, ..., 8.0. The triangle marks the CDI score of the CDI-selected label set among these candidate label sets; the star marks the CDI score of the layer 3 annotation provided in the previous study.

Global:

(GENES)	(CELLS)	[SEED]
5000	3000	1

Batches:

[BATCHES]	[BATCH CELLS]	[Location]	[Scale]
1	3000	0.1	0.1

Mean:

(Rate)	(Shape)
0.3	0.6

Library size:

(Location)	(SCALE)	(Norm)
11	0.1	FALSE

Exprs outliers:

(Probability)	(Location)	(Scale)
0.05	4	0.5

Groups:

[GROUPS]	[GROUP PROBS]
5	0.2, 0.2, 0.2, 0.2, 0.2

Diff expr:

[PROBABILITY]	[DOWN PROB]	[LOCATION]	[SCALE]
0.01, 0.01, 0.01, 0.01, 0.01	0	0.4	0.1

BCV:

(Common Disp)	(DoF)
0.1	60

Dropout:

[Type]	(Midpoint)	(Shape)
none	0	-1

Paths:

[From]	[Steps]	[Skew]	[Non-linear]	[Sigma Factor]
0	100	0.5	0.1	0.8

Fig. S17: Parameters used in the Splatter simulator to generate SD4.

2 Note 1

Notations

- c: index of cell ($c = 1, \dots, N$)
- g: index of gene ($g = 1, \dots, G$)
- k: index of cell-type ($k = 1, \dots, K$)
- X_{gc}: raw UMI count for gene g in cell c
- s_c: size factor for cell c, defined below in the size factor estimation.
- ϕ: dispersion parameter shared by all genes and all cells in one dataset
- δ: zero inflation factor shared by all genes and all cells in one dataset
- μ_g: mean parameter for gene g across all cells
- ϕ_g: dispersion parameter for gene g across all cells
- δ_g: zero-inflation factor for gene g across all cells
- $\mu_{g,k}$: mean parameter for gene g in cell-type k
- $\phi_{g,k}$: dispersion parameter for gene g in cell-type k
- $\delta_{g,k}$: zero-inflation factor for gene g in cell-type k

The size factor s_c is a robust estimator of cell libary size. Its calculation follows the DESeq2 median-of-ratios procedure.

$$s_c = \text{median}_g \frac{\max\{X_{gc}, 0.5\}}{\left(\prod_{c=1}^{N} \max\{X_{gc}, 0.5\}\right)^{1/N}}.$$

We add 0.5 to zero count to avoid the zero denominators.

Model distributions

First, we define the probability mass function (pmf) of NB(μ, ϕ) distribution. For any $Y \sim \text{NB}(\mu, \phi)$ and $y \in \mathbb{N} \cup \{0\}$,

$$P(Y = y) = \frac{\Gamma(y + 1/\phi)}{\Gamma(y+1)\Gamma(1/\phi)} \left(\frac{1/\phi}{\mu + 1/\phi}\right)^{1/\phi} \left(\frac{\mu}{\mu + 1/\phi}\right)^{y} (\mu > 0, \phi > 0).$$

Next, we define the pmf of ZINB(μ, ϕ, δ) distribution. For any $Y \sim \text{ZINB}(\mu, \phi, \delta)$ and $y \in \mathbb{N} \cup \{0\}$,

$$P(Y = y) = \begin{cases} \delta \left(\frac{1/\phi}{\mu+1/\phi}\right)^{1/\phi}, & \text{if } y = 0; \\ (1-\delta)\frac{\Gamma(y+1/\phi)}{\Gamma(y+1)\Gamma(1/\phi)} \left(\frac{1/\phi}{\mu+1/\phi}\right)^{1/\phi} \left(\frac{\mu}{\mu+1/\phi}\right)^{y}, & \text{if } y > 0. \end{cases} \quad (\mu > 0, \phi > 0, \delta \in [0, 1))$$

In the manuscript, the models we considered are listed in Table. S1.

Model name	Distribution of X_{gc}
Gene-common NB	$\mathrm{NB}(s_c\mu_g, \phi)$
Gene-common ZINB	$\mathrm{ZINB}(s_c\mu_g, \phi, \delta)$
Gene-specific NB	$\mathrm{NB}(s_c\mu_g, \phi_g)$
Gene-specific ZINB	$\mathrm{ZINB}(s_c\mu_g, \phi_g, \delta_g)$
Cell-type-common NB	$\mathrm{NB}(s_c\mu_g, \phi_g)$
Cell-type-common ZINB	$\mathrm{ZINB}(s_c\mu_g, \phi_g, \delta_g)$
Cell-type-specific NB	$\mathrm{NB}(s_c\mu_{g,k}, \phi_{g,k})$ for cell c in cell-type k
Cell-type-specific ZINB	$\mathrm{ZINB}(s_c\mu_{g,k}, \phi_{g,k}, \delta_{g,k})$ for cell c in cell-type k

Table. S1: Model names and the corresponding distributions of raw UMI count.

3 Note 2

For simplicity we omit the batch index b in the superscript (if any) and size factor s_c. Suppose

$$L_{0,c} \sim \text{Multinomial}(K_0; \pi_1, \ldots, \pi_{K_0}).$$

Combining with the NB model

$$X_{gc} \mid (L_{0,c} = k) \sim \mathrm{NB}(\mu_{gk}, \phi_{gk}),$$

we have $X_{gc} \sim \sum_{k=1}^{K_0} \pi_k \mathrm{NB}(\mu_{gk}, \phi_{gk})$ with

$$\xi_{1,g} = \mu_g = \mathtt{E}(X_{gc}) = \sum_{k=1}^{K_0} \pi_k \mu_{gk}, \quad \xi_{2,g} = \mu_g^2 + \sigma_g^2 = \mathtt{E}(X_{gc}^2) = \sum_{k=1}^{K_0} \pi_k \{\mu_{gk} + (1+\phi_{gk})\mu_{gk}^2\}.$$

Therefore, $\xi_{2,g} - \xi_{1,g} = \sum_{k=1}^{K_0} \pi_k (1+\phi_{gk})\mu_{gk}^2$. Let $\xi_{3,g} = \sum_{k=1}^{K_0} \pi_k (1+\phi_{gk})^{1/2}\mu_{gk}$. Then

$$\eta_g = \frac{\xi_{2,g} - \xi_{1,g}}{\xi_{3,g}^2} = \sum_{k=1}^{K_0} \pi_k \left\{ \frac{(1+\phi_{gk})^{1/2}\mu_{gk}}{\xi_{3,g}} \right\}^2.$$

Let

$$a_k = \pi_k^{1/2} \frac{(1+\phi_{gk})^{1/2}\mu_{gk}}{\xi_{3,g}}, \quad b_k = \pi_k^{1/2}.$$

By $a_k^2 \geq 2a_k b_k - b_k^2$, we have

$$\eta_g = \sum_{k=1}^{K_0} a_k^2 \geq 2\sum_{k=1}^{K_0} a_k b_k - \sum_{k=1}^{K_0} b_k^2 = 2\sum_{k=1}^{K_0} \frac{\pi_k (1+\phi_{gk})^{1/2}\mu_{gk}}{\xi_{3,g}} - \sum_{k=1}^{K_0} \pi_k = 1.$$

The minimum is obtained when $a_k = b_k$ for all $k \in \{1, \ldots, K_0\}$, *i.e.*,

$$(1+\phi_{gk})\mu_{gk}^2 = C_g, \tag{1}$$

not depending on the cell type k.

If gene g is not a feature gene, then $\mu_{gk} = \mu_g$ and $\phi_{gk} = \phi_g$, thus (1) holds, and η_g will reach the minimum 1.

If gene g is a feature gene, then as long as $(1+\phi_{gk})\mu_{gk}^2$ are not all equal across cell type k, $\eta_g > 1$. Thus for feature genes, η_g tends to be large.

Unfortunately, $\xi_{3,g}$ is hard to estimate if the true cell labels are unknown. Thus, η_g is hard to estimate directly. We need to find a surrogate ξ_g to approximate η_g.

It is easy to see that the WDS for gene g is

$$\xi_g = \frac{\sigma_g^2 - \mu_g}{\mu_g^2} = \frac{\xi_{2,g} - \xi_{1,g}^2 - \xi_{1,g}}{\xi_{1,g}^2} = \frac{\xi_{2,g} - \xi_{1,g}}{\xi_{1,g}^2} - 1.$$

When $\phi_{gk} \approx \phi$ for all g and k, $\xi_{3,g} \approx (1+\phi)^{1/2}\xi_{1,g}$. Then, $\xi_g \approx \eta_g(1+\phi) - 1$.

In general, let $\phi_{\max} = \max_{k,g} \phi_{g,k}$. Then η_g can be bounded on both sides:

$$\frac{1}{1+\phi_{\max}}(\xi_g + 1) \leq \eta_g \leq \xi_g + 1.$$

We assume that $\phi_{\max}$ are not too large. Then ξ_g can be used as a surrogate of η_g. We estimate ξ_g by

$$\hat{\xi}_g = \frac{\hat{\sigma}_g^2 - \hat{\mu}_g}{\hat{\mu}_g^2}.$$
