# Supplementary material for: Clustering Deviation Index (CDI): a robust and accurate internal measure for evaluating scRNA-seq data clustering
Source: Genome Biol. 2022 Dec 27;23:269. doi: 10.1186/s13059-022-02825-5 (PMC9793368; doi:10.1186/s13059-022-02825-5)
Supplement: Supplementary file 2 — Additional file 2. Review history. [file 13059_2022_2825_MOESM2_ESM.docx]

Review History

**First round of review**

**Reviewer 1**

**Were you able to assess all statistics in the manuscript, including the appropriateness of statistical tests used?**

Yes

**Comments to author:**

The authors’ approach to judge the quality of clustering using unsupervised modeling of the count-distribution of the scRNA-seq data is interesting and novel. I have the following technical concerns. And the manuscript is written poorly in several places

Major comments:

(1)	First, Fig. 5 is mentioned before Fig. 1-4 (“None of their selected label sets matches the benchmark label set (Fig. 5).”). This is usually not good. The order of the figures or the order in which the figures are discussed needs to be adjusted. Second, I cannot understand the meaning of Fig. 5, without reading all the other parts of the manuscript first. I only came to understand this figure when I read the last result section. The authors need to explain this figure very carefully and pay attention to order.

(2)	“We built our model on a rigorous characterization of the statistical properties of the scRNA-seq data, CT26.WT, from a pure monoclonal population” Will your approach be not suitable for scRNA-seq data generated from other tissue types? Or different sequencing depth? Will low seq depth scRNA-seq datasets suffer more obvious zero-inflation (drop-outs?). I suggest the authors to test their idea on at least one other monoclonal dataset, ideally with lower sequencing depth

(3)	“To fit their UMI count distributions, we consider the following two NB distribution families.” It’s not clear to me how the parameters of the distribution are fitted to the real data with mixed cell types. I checked Sup Note 1, but there is still no such information. Specifically what technique was used? Is there a de-convolution estimation step involved?

(4)	It’s unclear to me how the different library sizes of the cells were adjusted for when calculating CDI. It appears that this has not been done. Isn’t there a need to do this? Let’s say there is a hypothetical situation where a cell’s sequencing depth is amplified by many fold. Then this artificial change (without normalization of library size) will skew the assumed distribution by the authors, leading to problematic estimates of likelihood.

(5)	The organization of the manuscript is poor in many places. For example, here: “CDI following VST selected the six-cluster label set generated by the spectral clustering (ARI=0.39);” There is no explanation of the methods used to generate the candidate labels here at all. The explanation happened a few paragraphs later in this section: “Performance of CDI in selecting the optimal label set”

(6)	“CDI-BIC performed very well on all three datasets. It selected the label sets with the correct numbers of clusters (Fig. 4A)”. I would probably disagree with the author regarding their interpretation. For the cortex dataset, CDI-BIC didn’t find the correct number of clusters (main), and on the retina dataset, CDI-AIC didn’t find the correct number of clusters (subtype)

(7)	“moreover, the selected label sets are very similar to the benchmark label sets (Fig. 4B).” The authors need to quantify this “similarity” in terms of cellular abundances and compare it across different methods, to show that CDI is really the best.

(8)	Fig 5 uses ARI as the gold-standard to judge the performance of the unsupervised methods. This seems a little cherry-picking. The authors need to use other supervised approaches to serve as the gold-standard, and do these analyses again.

Minor comment

(1)	“both UMAPs look similar (Fig. ??E).” I see this on my downloaded pdf. Same here: “(T-CELL panel in Fig. ??A, B).”

(2)	“normal-sized”. This wording is a little weird. Maybe find a better one? It’s hard to say that rare cell types are not normal

(3)	“An unsupervised index performs well if it is a monotone function of ARI.” Meaning not clear

**Reviewer 2**

**Were you able to assess all statistics in the manuscript, including the appropriateness of statistical tests used?**

No

**Comments to author:**

This paper proposed a new Clustering Deviation Index (CDI) that measures the deviation of any clustering label set from the observed single-cell data. They applied CDI to multiple experimental scRNA-seq data sets to demonstrate that it successfully selected biologically meaningful clustering labels in each case. Generally speaking, the novelty of this manuscript seems to be limited. Specifically, the following major concerns should be addressed.

1. In this paper, NB distribution is used to fit UMI count data. Can NB distribution also fit other types of count data? For example, FPKM count data.

2. In section "Background", the authors said that DCI's performance on scRNA-seq is consistent with ARI (Fig. 5). How is this reflected in Fig.5?

3. All figures are not very self-explaining. More details can be added to the figures and the captions.

4. In the experiment, only four real scRNA-seq datasets are used. To make your unsupervised clustering index CDI more convincing, you should use more datasets for testing.

5. Scalability is one of the essential requirements for scRNA-seq data clustering methods. The datasets used in this manuscript are too small. The authors should try their method on large-scale datasets (e.g., >10k, >50k, >100k, >200k, >500k single cells). If possible, trying on a million-cell dataset would be preferred.

**Reviewer 3**

**Were you able to assess all statistics in the manuscript, including the appropriateness of statistical tests used?**

Yes

**Comments to authors:**

The authors introduced a new clustering validation metric for selecting the "optimal" clustering partition from a collection of possible clustering partitions generated from any given clustering algorithms. The author labeled the metric as "unsupervised" given that the ground truth cluster labels of a dataset is not used for selection the "optimal" clustering output. Simulation and experimental datasets were used for comparing their proposed methods with alternative methods. We have the following main comments:

1. There is a lack of motivation for the proposed method. Clustering validation is a well-studied topic and a large number of clustering validation metrics have been proposed. Using the authors terminology (i.e. "unsupervised" and "supervised" method), there are more than 20 such "unsupervised" metrics for assessing clustering output (see for example: [1] https://cran.r-project.org/web/packages/clValid/vignettes/clValid.pdf and [2] https://cran.r-project.org/web/packages/clusterCrit/vignettes/clusterCrit.pdf). The key motivation question is why we need yet another clustering evaluation metric and what gap does it fill?

2. The presentation of the manuscript needs improvement and the literature on this research topic is mostly missed by the authors. In particular, categorizing clustering validation metrics into "unsupervised" and "supervised" indices is unconventional. The literature typically categorizes clustering evaluation metrics into "internal" and "external" metrics. There is also "stability" metrics commonly used with perturbation on data or tuning of clustering parameters. We wonder why the authors redefine these metrics into "unsupervised" and "supervised" indices and how do they relate to the literature? More broadly, it appears that the literature of clustering validation is missing in the background of the manuscript.

3. The comparison experiments conducted by the authors are quite limited. While there are a large number of internal metrics, the authors only compared to three methods (Silhouette coefficient, Calinski-Harabaz index and Davies-Bouldin index). It is difficult to conclude how much the proposed index further informs the field in terms of choosing internal indices for scRNA-seq data analysis given the comparison experiments conducted by the authors.

4. The attribution of external metrics (ARI, NMI, and FM) in the background section and the comparison experiments conducted by the authors are contradicting. In particular, the authors mentioned:

"… the benchmark label set ... Moreover, even if it is available, it could be biased or incorrect because of outdated domain knowledge, leading to poor performances of supervised indices."

Yet, the performance assessment is based on the concordance of the clustering output with the ground truth quantified by ARI. How does this align with the authors statement of "supervised indices" may be poor?

Additional issues:

5. Figure 4 panel A shows that the CDI can choose the best number of clusters, so what is the result of Davies-Bouldin index, Silhouette coefficient, and Calinski-Harabasz index for pick up the number of clusters on those datasets?

6. Current performance comparisons rely alone on ARI (Figure 5). What would the results look like by using other external metrics? How would the methods fare on alternative criteria such as selecting the optimal number of clusters from a given dataset?

7. The authors concluded that common NB underestimates the zero UMI count proportion but gene-specific NB yielded reasonable zero UMI count proportions (in Page 4 line 41 to line 48). However, in Figure 1A, the two figures lie in the first row (Gene-common NB and Gene-specific NB) are the same.

8. Page2 line 49, "if they are similar, ARI is close to 1; otherwise close to 0." Adjusted Rand index can yield negative values if the index is less than the expected index.

9. Page 17 line 20, Catesian -> Cartesian

**Authors Response**

**Point-by-point responses to the reviewers’ comments:**

The authors’ approach to judge the quality of clustering using unsupervised modeling of the count-distribution of the scRNA-seq data is interesting and novel. We thank the reviewer for the summary and appreciation of our work. I have the following technical concerns. And the manuscript is written poorly in several places.

Major comments:

1) First, Fig. 5 is mentioned before Fig. 1-4 (“None of their selected label sets matches the benchmark label set (Fig. 5).”). This is usually not good. The order of the figures or the order in which the figures are discussed needs to be adjusted. Second, I cannot understand the meaning of Fig. 5, without reading all the other parts of the manuscript first. I only came to understand this figure when I read the last result section. The authors need to explain this figure very carefully and pay attention to order.

*We revised the introduction to improve the logic flow and the reading experience. The figures are now logically placed to support the development in the text. We also improved the presentation of the figures. The original Figure 5 has been replaced and now provides a comprehensive comparison of all internal indices, including the cluster numbers in their selected label sets, the quality of the selected label sets, and their Spearman correlation with the more accurate external indices.*

2) “We built our model on a rigorous characterization of the statistical properties of the scRNA-seq data, CT26.WT, from a pure monoclonal population”. Will your approach be not suitable for scRNA-seq data generated from other tissue types? Or different sequencing depth? Will low seq depth scRNA-seq datasets suffer more obvious zero-inflation (dropouts?). I suggest the authors test their idea on at least one other monoclonal dataset, ideally with a lower sequencing depth.

*In our paper, by saying “UMI counts are not zero-inflated, we mean that “the UMI counts do not contain excessive zeros compared with the negative binomial distributions”. This still allows the UMI counts to have many zero observations. Many previous studies supported the conclusion. These studies checked the UMI distributions in multiple monoclonal datasets from multiple tissue types and different sequencing depths, and all found that the UMI counts were not zero-inflated. [1, 2, 3, 4]. Specifically, [2] used eight public datasets and concluded that high-throughput droplet-based methods that make use of UMI counts are not zero-inflated; [4] further clarified that UMI counts are not zero-inflated even for low-throughput plate-based methods. However, for those sequencing protocols with no UMI counts but only read counts, including the PCR replicates, the read counts are more zero-inflated than the negative binomial distribution. The PCR replicates elevated the non-zero read counts and distorted the distributions. Thus, our model and method do not apply to the read counts with the PCR replicates. We have incorporated this review of previous studies on the zero-inflation problem to page 5 lines 104-117.*

*Notably, although several studies have checked that the UMI counts are not inflated in multiple monoclonal datasets, the monoclonal datasets they used were generated by either spiking in the ERCC RNA or purifying cells by fluorescence-activated cell sorting. None of them used the monoclonal cell populations from cell cloning. We explained the benefit of using cell cloning for model evaluation in page 19 lines 505-523. Besides, we also compared different NB families to find the most fitted NB family for the UMI counts. Details are in page 4 line 81 - page 5 line 103.*

3) “To fit their UMI count distributions, we consider the following two NB distribution families.” It’s not clear to me how the parameters of the distribution are fitted to the real data with mixed cell types. I checked Sup Note 1, but there is still no such information. Specifically what technique was used? Is there a de-convolution estimation step involved?

*We did not use any de-convolution method here. First, the polyclonal datasets T-CELL, CORTEX and RETINA have benchmark label sets indicating their cell types. These benchmark label sets were “obtained by the experimental and bioinformatics analysis process, including fluorescence-activated cell sorting (FACS), known feature gene checking, cell screening, and clustering; thus, these benchmark label sets may not be accurate but reflect our best knowledge of the cell types.” (Table 1 caption) Similar explanations are also provided in the page 8 line 212 - page 9 line 225.*

*Second, to fit the UMI count distribution, we partitioned the cells into different cell types based on the benchmark label set. Within each cell type and each gene, we fit a negative binomial distribution and estimated their mean and dispersion parameters with maximum likelihood estimators. We did two analyses to evaluate the model fit: 1) We split the data into two halves for training and test and compared the observed zero proportion in the test data with the estimated zero proportion in the training data; 2) We applied Cell-type-specific “goodness-of-fit” tests as described in page 23 line 617 - page 24 line 638.*

4) It’s unclear to me how the different library sizes of the cells were adjusted for when calculating CDI. It appears that this has not been done. Isn’t there a need to do this? Let’s say there is a hypothetical situation where a cell’s sequencing depth is amplified by many folds. Then this artificial change (without normalization of library size) will skew the assumed distribution by the authors, leading to problematic estimates of likelihood.

*We agree with the reviewer that adjusting for the library size when calculating CDI is very important. In fact, we used the size factor s c as a coefficient of the mean parameter in negative binomial distribution to adjust for the difference in cell library size. Each cell has its own size factor value, and the definition of sc follows the DESeq2 median-of-ratios procedure [5].*

*To clarify that our models have been adjusted for the cellular library size, we added more explanations when we introduced the models in Results: UMI count distributions. In the page 4 lines 86-88, we mentioned that “These models have adjusted for the cell library size via a size factor sc (Supplementary Note 1).” In page 6 line 152, we re-emphasized that “Notably, these models have adjusted for the cell library size”. The details of sc definition were provided in Supplementary Note 1*

5) The organization of the manuscript is poor in many places. For example, here: “CDI following VST selected the six-cluster label set generated by the spectral clustering (ARI=0.39);” There is no explanation of the methods used to generate the candidate labels here at all. The explanation happened a few paragraphs later in this section: “Performance of CDI in selecting the optimal label set”.

*As suggested, we re-organized the structure of the paper. At the beginning of Results: Performance Evaluation, we added the explanations of all the datasets and the clustering methods we used. Now, these paragraphs are placed before the paragraph where we discussed the Performance of WDS. Details are in the page 8 line 209 - page 9 line 236.*

6)“CDI-BIC performed very well on all three datasets. It selected the label sets with the correct numbers of clusters (Fig. 4A)”. I would probably disagree with the author regarding their interpretation. For the cortex dataset, CDI-BIC didn’t find the correct number of clusters (main), and on the retina dataset, CDI-AIC didn’t find the correct number of clusters (subtype).

*As suggested, we clarified our expressions in the following way.*

*• We clarified in page 11 line 294 - 297 that “ CDI-BIC selected the label sets with the correct numbers of clusters for datasets without rare cell types (SD1, SD4, T-CELL panels in Fig. 4A); moreover, the selected label sets are similar to the benchmark label sets (SD1, SD4, T-CELL panels in Fig. 4B).”*

*• We clarified in page 15 lines 415-418 that “For CORTEX and RETINA, the CDIselected label sets have more clusters than the benchmark label set: the selected label set is similar to a finer partition of the benchmark label set.” In page 16 line 424, we pointed out that “However, the number clusters of the selected label sets do not fully reflect the quality of the selected label set.”*

*• To evaluate the quality of the optimal label set selected by various internal indices, we compared these label sets with the benchmark label sets by external indices: Adjusted Rand Index (ARI), Fowlkes-Mallows Scores (FM), and Normalized Mutual Information (NMI).” These external indices use benchmark cell type labels and are more accurate than internal metrics that do not use benchmark cell type information. Details of these external metrics can be found in page 15 Table 2. The result in manuscript Fig. 5B (copied in this document) showed that the CDI selected label set obtained the highest median ARI, FM, and NMI values among nine internal metrics. More specifically, for CORTEX benchmark main type and subtype labels, the CDI selected label set achieved the highest ARI, FM, and NMI values among nine internal metrics. For RETINA main type labels, the CDI selected label set are among the top two highest ARI, FM, and NMI values. For RETINA benchmark subtypes, CDI selected label set didn’t achieve a high ARI or FM values, but it ranked second for NMI value. More details are in page 15, Results: Comparison of CDI with other internal indices.*

7) “Moreover, the selected label sets are very similar to the benchmark label sets (Fig. 4B).” The authors need to quantify this “similarity” in terms of cellular abundances and compare it across different methods, to show that CDI is really the best.

*As suggested, we added the cellular abundances to quantify the similarity between cellular abundance between the CDI-selected label set and the benchmark label set. In page 11 lines 297-310, we added “For SD4, CDI selected the label set identical to the benchmark label set; for SD1 and T-CELL, CDI selected the label set with 98% and 95% of the cells correctly allocated to their benchmark clusters.” If the selected label set has the same number of clusters as the benchmark label set, it is possible to quantify their similarity by matching the clusters based on majority votes and then checking the proportions of the cells that are assigned to the matching clusters. These cells are defined as the “correctly assigned” cells. However, when the selected label set has more or fewer clusters, it is challenging to define the “correctly assigned cells”. Thus, we need to use other approaches to quantify the similarity between the selected label and the benchmark label. We used three external indices (Adjusted Rand Index (ARI), FowlkesMallows Scores (FM), and Normalized Mutual Information (NMI)) to measure the quality of the label sets. Details are in page 15, Results: Comparison of CDI and other internal indices.*

8) Fig 5 uses ARI as the gold standard to judge the performance of the unsupervised methods. This seems a little cherry-picking. The authors need to use other supervised approaches to serve as the gold standard and do these analyses again.

*As suggested, in this revision, we included another two external metrics, Fowlkes-Mallows Scores (FM) and Normalized Mutual Information (NMI), together with Adjusted Rand Index (ARI) as the gold standard to evaluate the performance of internal metrics.*

*We also provided more comprehensive comparisons between CDI and another eight internal indices (Manuscript Fig. 5, copied in this document). First, we compared the number of clusters of the optimal label set selected by different internal indices (Manuscript Fig. 5A). Second, we calculated the ARI, FM, and NMI of the label sets selected by various internal indices (Manuscript Fig. 5B). The CDI-selected label sets have the largest median ARI, FM, and NMI across datasets compared with the label sets selected by other internal indices. Third, we calculated the Spearman correlations between the internal indices and external indices. CDI has the highest Spearman correlations with ARI, FM, and NMI (Manuscript Fig 5C). Details are in page 15, Results: Comparison of CDI with other internal indices.*

Minor comment: 1) “both UMAPs look similar (Fig. ??E).” I see this on my downloaded pdf. Same here: “(T-CELL panel in Fig. ??A, B).”

*We corrected these typos.*

2) “normal-sized”. This wording is a little weird. Maybe find a better one? It’s hard to say that rare cell types are not normal.

*We changed “normal-sized” to “abundant”.*

3) “An unsupervised index performs well if it is a monotone function of ARI.” Meaning not clear

*In the revised manuscript, we now use the Spearman correlations to measure the agreement between internal indices and external indices. Details are in page 16 line 436-454.*

*Manuscript Fig 5: Comparison between CDI and other internal indices. (A) The barplot shows the cluster number of the benchmark label set and the label sets selected by different internal indices. Each panel compares the cluster number of a benchmark label set with all other selected label sets for one dataset. For the dataset with both main type and subtype benchmark label sets (SD3, CORTEX, and RETINA), the panel with the subtype benchmark label set is listed with “(subtype)”. (B) The boxplot shows the external indices of the label sets selected by different internal indices. The external indices are calculated by comparing the selected label sets and the benchmark label sets. (C) The boxplot shows the Spearman correlation between the internal and external indices while ranking all candidate label sets. The dots in (B) and (C) mark the external indices or Spearman correlations for main type benchmark label sets. The triangles mark the external indices or Spearman correlations for subtype benchmark label sets.*

Reviewer #2:

This paper proposed a new Clustering Deviation Index (CDI) that measures the deviation of any clustering label set from the observed single-cell data. They applied CDI to multiple experimental scRNA-seq data sets to demonstrate that it successfully selected biologically meaningful clustering labels in each case. Generally speaking, the novelty of this manuscript seems to be limited.

*We thank the reviewer for the summary of our method.*

Specifically, the following major concerns should be addressed.

1) In this paper, NB distribution is used to fit UMI count data. Can NB distribution also fit other types of count data? For example, FPKM count data.

*We tested whether the NB distributions fit the FPKM and TPM normalized count data showed the testing results in Fig. S1B and Fig. S1C (copied in this document).We also added the explanations in page 6 line 138, “The gene-specific NB family is only designed to fit the raw UMI counts of the monoclonal cells; it fits poorly on the distribution of the normalized counts (FPKM in Supplementary Fig. S1B, TPM in Supplementary Fig. 1C). When the test type I error is 5%, the gene-common NB, gene-common ZINB, and gene-specific NB families do not fit the normalized count distributions for almost all genes; the gene-specific ZINB family does not fit the normalized count distributions for more than 30% of the genes.”*

2) In section “Background”, the authors said that CDI’s performance on scRNA-seq is consistent with ARI (Fig. 5). How is this reflected in Fig.5?

*Following multiple reviewers’ suggestions, we changed the comparison frameworks and added more internal indices and external indices (Adjusted Rand Index (ARI), FowlkesMallows Scores (FM), and Normalized Mutual Information (NMI)). We provided more comprehensive comparisons between CDI and another eight internal indices (Manuscript Fig. 5, copied in this document). First, we compared the number of clusters of the optimal label set selected by different internal indices (Manuscript Fig. 5A). Second, we calculated the ARI, FM, and NMI of the label sets selected by various internal indices (Manuscript Fig. 5B). The CDI-selected label sets have the largest median ARI, FM, and NMI across datasets compared with the label sets selected by other internal indices. Third, we calculated the Spearman correlations between the internal indices and external indices. CDI has the highest Spearman correlations with ARI, FM, and NMI (Manuscript Fig 5C). Details are in page 15, Results: Comparison of CDI with other internal indices.*

3) All figures are not very self-explaining. More details can be added to the figures and the captions.

*We provided more details in the caption of all figures.*

4) In the experiment, only four real scRNA-seq datasets are used. To make your unsupervised clustering index CDI more convincing, you should use more datasets for testing.

5) Scalability is one of the essential requirements for scRNA-seq data clustering methods. The datasets used in this manuscript are too small. The authors should try their method on large-scale datasets (e.g., > 10k, > 50k, > 100k, > 200k, > 500k single cells). If possible, trying on a million-cell dataset would be preferred.

*These two suggestions are related, and thus we would like to address them together. Following the suggestions, we added two large-scale datasets: IPF (114,396 cells) and COVID (1,251,200) cells. The CDI results of these two large-scale datasets are shown in page 13 line 371 - page 14 line 395.*

*To show CDI’s computation scalability, we added a new section in page 17, Results: Computation time. We compared the computation time of CDI and other internal indices and found that CDI is the fastest. More importantly, for very large-scale datasets like COVID (with more than 1 million cells), CDI is the only computationally feasible internal index that can select the optimal label set. More details can be found in Manuscript Fig 6 (copied in this document).*

*Supplementary Fig S1: Percentages of genes rejected by the Pearson “goodness-offit” tests in CT26.WT when the type I errors were set at 0.5%, 1%, 5%, and 10%. We checked the distributions of the raw UMI counts (A), the FPKM normalized counts (B), and the TPM normalized counts (C). The gene length for calculating FPKM and TPM was obtained from R package EDASeq by mapping the gene emsembl IDs to the external database.*

Manuscript Fig 6: Computation time of nine internal indices. The computation time of all internal indices was evaluated on the five experimental datasets based on 500 selected genes and benchmark cell type labels. The x-axis labels the cell numbers in thousands of these datasets. CDI allows considering batch effect. The triangle corresponds to CDI calculation considering the batch effect, and the dot corresponds to calculating CDI without batch effect. The y-axis labels the computation time in the log10 scale. Algorithms of indices were tested on one Intel(R) Xeon(R) Gold 6252 CPU.

Reviewer #3:

The authors introduced a new clustering validation metric for selecting the “optimal” clustering partition from a collection of possible clustering partitions generated from any given clustering algorithm. The author labeled the metric as “unsupervised” given that the ground truth cluster labels of a dataset are not used for selecting the “optimal” clustering output. Simulation and experimental datasets were used for comparing their proposed methods with alternative methods.

*We thank the reviewer for providing the summary.*

We have the following main comments:

1) There is a lack of motivation for the proposed method. Clustering validation is a wellstudied topic and a large number of clustering validation metrics have been proposed. Using the authors’ terminology (i.e. “unsupervised” and “supervised” method), there are more than 20 such “unsupervised” metrics for assessing clustering output (see for example: [1] https://cran.r-project.org/web/packages/clValid/vignettes/clValid.pdf and [2] https://cran.r-project.org/web/packages/clusterCrit/vignettes/clusterCrit.pdf). The key motivation question is why we need yet another clustering evaluation metric and what gap does it fill?

*As suggested, the motivation of the study has been clarified and expanded in the introduction of the manuscript. We aim to develop a robust, computationally efficient, and accurate internal index to select the optimal clustering label set that fits the scRNA-seq data. There are many existing internal indices available, as suggested by the reviewer. However, none of these indices are specifically designed to select the optimal label set for scRNA-seq data. In contrast, CDI is designed explicitly for scRNA-seq data clustering. Because CDI utilizes the distribution of the UMI counts in scRNA-seq data, it is expected to perform better than the other general internal indices for scRNA-seq UMI counts.*

*In this revision, we included eight internal indices to compare with CDI and three external indices (Adjusted Rand Index (ARI), Fowlkes-Mallows Scores (FM), and Normalized Mutual Information (NMI)) as the gold standard. We provided more comprehensive comparisons between CDI and another eight internal indices (Manuscript Fig. 5, copied in this document). First, we compared the number of clusters of the optimal label set selected by different internal indices (Manuscript Fig. 5A). Second, we calculated the ARI, FM, and NMI of the label sets selected by various internal indices (Manuscript Fig. 5B). The CDI-selected label sets have the largest median ARI, FM, and NMI across datasets compared with the label sets selected by other internal indices. Third, we calculated the Spearman correlations between internal and external indices. CDI has the highest Spearman correlations with ARI, FM, and NMI (Manuscript Fig 5C). Details are in page 15, Results: Comparison of CDI with other internal indices. These results show that CDI has consistently better performance than other internal indices. Besides, it is also the most computationally efficient index (Manuscript Fig 6, copied in this document). Details are in page 17, Results: Computation time.*

*The clusterCrit package contains more than 20 internal metrics. We applied eight of them that are listed as popular metrics in the review paper on internal metrics[6]. In these eight metrics, “S Dbw” cannot handle sparse data like scRNA-seq UMI counts and generated NANs for almost all datasets. We excluded “S Dbw”. We included seven internal metrics calculated from clusterCrit in our manuscript. The clValid package has three types of metrics: the “biological” metric is for gene clustering; the “stability” metric does not fit our study aim (as explained in page 2 line 44 - page 3 line 59). Thus, we only used the internal metric “connectivity”. The other two internal metrics mentioned in clValid (Silhouette Width and Dunn Index) were included in the clusterCrit package.*

2) The presentation of the manuscript needs improvement and the literature on this research topic is mostly missed by the authors. In particular, categorizing clustering validation metrics into “unsupervised” and “supervised” indices is unconventional. The literature typically categorizes clustering evaluation metrics into “internal” and “external” metrics. There are also “stability” metrics commonly used with perturbation of data or tuning of clustering parameters. We wonder why the authors redefine these metrics into “unsupervised” and “supervised” indices and how do they relate to the literature? More broadly, it appears that the literature of clustering validation is missing in the background of the manuscript.

*Here are our changes following the suggestion.*

*• We changed “unsupervised” to “internal” and “supervised” to “external” to meet the convention.*

*• The aim of our study is to develop an internal index that can select the optimal label set that fits the scRNA-seq data. We clarified it in page 3 line 60 - 72.*

*• We reviewed and cited the literature of internal and external clustering validation metrics in page 2 line 22-43. Specially, in the page 2 line 44 - page 3 line 59, we added a review for the stability indices and explained why these indices do not fit our study aim.*

3) The comparison experiments conducted by the authors are quite limited. While there are a large number of internal metrics, the authors only compared to three methods (Silhouette coefficient, Calinski-Harabaz index and Davies-Bouldin index). It is difficult to conclude how much the proposed index further informs the field in terms of choosing internal indices for scRNA-seq data analysis given the comparison experiments conducted by the authors.

*As answered in question 1), besides Silhouette coefficient, Calinski-Harabaz index and Davies-Bouldin index, we now included five more internal indices in the comparison (listed in page 15 Table 2). We compared CDI and their performance in selecting the optimal label set (Manuscript Fig. 5C, copied in this document). Details are in page 15, Results: Comparison of CDI with other internal indices. We also compared their computation time (Manuscript Fig. 6, copied in this document). Details are in page 16, Results: Computation time.*

4) The attribution of external metrics (ARI, NMI, and FM) in the background section and the comparison experiments conducted by the authors are contradicting. In particular, the authors mentioned: “. . . the benchmark label set ... Moreover, even if it is available, it could be biased or incorrect because of outdated domain knowledge, leading to poor performances of supervised indices.” Yet, the performance assessment is based on the concordance of the clustering output with the ground truth quantified by ARI. How does this align with the authors’ statement of “supervised indices” may be poor?

*We would like to clarify the following points.*

*• In page 2 line 28, we clarified that “External indices utilize the benchmark label set to evaluate the quality of the candidate label set. If a high-quality benchmark label set is available, external indices are more accurate than internal indices, which do not utilize the high-quality benchmark label set. However, external indices cannot be applied when the benchmark label set is not available. Moreover, external indices may perform unsatisfyingly when the benchmark label set is of low quality. Both scenarios could occur for scRNA-seq clustering.”*

*• At the beginning of Results: Performance Evaluation, we clarified that some experimental datasets have high-quality benchmark label sets while others do not. The large-scale scRNA-seq datasets usually do not have high-quality benchmark label sets because annotating the large-scale scRNA-seq datasets is very challenging. The large-scale cell population is often highly heterogeneous with hierarchical cell structures; thus, generating reliable clustering results is difficult. Also, traditional verification approaches like manually checking gene markers is too laborious for large-scale datasets. For three moderate-scale datasets T-CELL (2,989 cells), CORTEX (7,390 cells), and RETINA (26,830 cells), the benchmark label sets were obtained by the experimental and bioinformatics analysis process, including fluorescence-activated cell sorting (FACS), known feature gene checking, cell screening, and clustering (Methods: Experimental datasets). These benchmark label sets are of high quality and use rigorous experimental methods and current bioinformatics best practices to assign cell labels. For the two large-scale datasets IPF (114,696 cells) and COVID (1,251,200 cells), the benchmark label sets might not be as reliable. More details are in Results: Performance of CDI.”*

Additional issues:

1) Figure 4 panel A shows that the CDI can choose the best number of clusters, so what is the result of Davies-Bouldin index, Silhouette coefficient, and Calinski-Harabasz index for pick up the number of clusters on those datasets?

*We now compared the cluster numbers of the label sets selected by nine different internal indices, including CDI. Details are in page 15 line 409 - page 16 line 423.*

2) Current performance comparisons rely alone on ARI (Figure 5). What would the results look like by using other external metrics? How would the methods fare on alternative criteria such as selecting the optimal number of clusters from a given dataset?

*We made the following changes.*

*• We included three external indices, Adjusted Rand Index (ARI), Fowlkes-Mallows Scores (FM), and Normalized Mutual Information (NMI).*

*• We provided three different criteria to evaluate the internal indices.*

*i) The cluster number of the optimal label set selected by the internal indices*

*ii) The external index scores of the optimal label set selected by the internal indices*

*iii) The Spearman correlation between the internal indices and the external indices.*

*The comparisons show that CDI has superior performance. The details are shown in page 15, Results: Comparison of CDI with other internal indices.*

3) The authors concluded that common NB underestimates the zero UMI count proportion but gene-specific NB yielded reasonable zero UMI count proportions (in Page 4 line 41 to line 48). However, in Figure 1A, the two figures that lie in the first row (Gene-common NB and Gene-specific NB) are the same.

*We apologize for mislabeling the panels in Figure 1A. The two panels in the first row should be Gene-common NB and Gene-common ZINB. These two panels look similar because the UMI count distributions do not have excessive zeros.*

4) Page2 line 49, “if they are similar, ARI is close to 1; otherwise close to 0.” Adjusted Rand index can yield negative values if the index is less than the expected index.

*We removed the inaccurate description and included more details of the three internal indices, ARI, FM, and NMI in page 15 Table 2.*

5) Page 17 line 20, Catesian − > Cartesian

*We corrected it.*

*References*

*[1] F William Townes, Stephanie C Hicks, Martin J Aryee, and Rafael A Irizarry. Feature selection and dimension reduction for single-cell RNA-Seq based on a multinomial*

*model. Genome Biol, 20(1):1–16, 2019.*

*[2] Valentine Svensson. Droplet scRNA-seq is not zero-inflated. Nat Biotechnol, 38(2):*

*147–150, 2020.*

*[3] Tae Hyun Kim, Xiang Zhou, and Mengjie Chen. Demystifying ”drop-outs” in single-cell*

*umi data. Genome Biol, 21(1):196, 08 2020. doi: 10.1186/s13059-020-02096-y.*

*[4] Yingying Cao, Simo Kitanovski, Ralf K¨uppers, and Daniel Hoffmann. Umi or not umi,*

*that is the question for scrna-seq zero-inflation. Nat. Biotechnol., 39(2):158–159, 2021.*

*[5] Michael I Love, Wolfgang Huber, and Simon Anders. Moderated estimation of fold*

*change and dispersion for RNA-seq data with deseq2. Genome Biol, 15(12):1–21, 2014.*

*[6] Yanchi Liu, Zhongmou Li, Hui Xiong, Xuedong Gao, and Junjie Wu. Understanding of*

*internal clustering validation measures. In 2010 IEEE international conference on data*

*mining, pages 911–916. IEEE, 2010.*

**Second round of review**

**Reviewer 1**

I thank the authors for answering all my questions!

I have only one minor concern: “An internal index is better if it ranks all candidate label sets more similarly than the external indices”. Do you actually mean “An internal index is better if it ranks all candidate label sets more similarly to the external indices”? Or do I miss something here?

Finally, it would be great if the authors could ask a native speaker or language editing service to help improve the language. The English is abstruse in places, though the scientific content is not compromised.

**Reviewer 2**

This paper can be accepted since the authors have revised it according to the comments.

**Reviewer 3**

The authors have addressed to each of my comment. However, the experimental setup is probably not permissive for an accurate assessment of the proposed CDI metric. In particular, my understanding of CDI is for estimating the number of clusters in a scRNA-seq dataset, yet the performance assessment is based on the clustering concordance to cell type label as defined in their original studies. We note that better performance on number of cluster estimation does not necessarily lead to better clustering concordance and vice versa (PMID: 35135612). Therefore, using the performance on clustering concordance to cell type label to assess the performance of CDI in the number of cluster estimation is not always informative. Furthermore, the reliance on external metrics (e.g., ARI) as gold standards to assess internal metrics (including the proposed CDI) can be problematic because it assumes that external metrics are always more accurate than internal metrics.

**Authors Response**

**Point-by-point responses to the reviewers’ comments:**

Reviewer 1

I thank the authors for answering all my questions!

*We thank the reviewer for their helpful comments that have improved the manuscript.*

I have only one minor concern: “An internal index is better if it ranks all candidate label sets more similarly than the external indices”. Do you actually mean “An internal index is better if it ranks all candidate label sets more similarly to the external indices”? Or do I miss something here?

*This is a typo. Thanks for pointing it out. We have corrected it. See line 437 of page 16.*

*Finally, it would be great if the authors could ask a native speaker or language editing service to help improve the language. The English is abstruse in places, though the scientific content is not compromised.*

*As suggested, we have requested a native speaker to improve the language. We hope the new version reads better.*

*Reviewer 2*

This paper can be accepted since the authors have revised it according to the comments.

*We thank the reviewer for their helpful comments that have improved the manuscript.*

*Reviewer 3*

The authors have addressed to each of my comment. However, the experimental setup is probably not permissive for an accurate assessment of the proposed CDI metric. In particular, my understanding of CDI is for estimating the number of clusters in a scRNA-seq dataset, yet the performance assessment is based on the clustering concordance to cell type label as defined in their original studies. We note that better performance on number of cluster estimation does not necessarily lead to better clustering concordance and vice versa (PMID: 35135612). Therefore, using the performance on clustering concordance to cell type label to assess the performance of CDI in the number of cluster estimation is not always informative. Furthermore, the reliance on external metrics (e.g., ARI) as gold standards to assess internal metrics (including the proposed CDI) can be problematic because it assumes that external metrics are always more accurate than internal metrics.

*We thank the reviewer for providing an opportunity to clarify the intended usage of CDI and how to evaluate its performance.*

*First, we would like to clarify that CDI is not an index for estimating the number of clusters. Rather, it measures the quality of the clustering label set, which encompasses the assignment of individual data points, and not just the cluster number. We completely agree with the conclusion in the existing literature (PMID: 35135612): better estimation of cluster numbers does not necessarily lead to better clustering concordance and vice versa. In fact, this insightful conclusion is reinforced in our experiments with CDII. On line 300 of page 11, which states:*

*“Notably, some candidate label sets have the correct numbers of clusters, but their cell labels are very different from the benchmarks. For example, for T-CELL, the two label sets with the lowest CDI are the five-cluster label sets generated by Seurat and SC3. These two label sets have similarly low CDIs (1.2744*$\times$*10^6^ for Seurat and 1.2743*$\times$*10^6^ for SC3) and similarly high ARIs (0.875 for Seurat and 0.870 for SC3). However, the five-cluster label sets generated by other methods have much higher CDIs (1.3356*$\times$*10^6^, 1.3095*$\times$*10^6^, 1.2918*$\times$*10^6^, 1.2812*$\times$*10^6^) and much lower ARIs (0.002, 0.116, 0.132, and 0.388). Thus, the label sets with lower CDIs have higher ARIs. The heatmaps also verified that the label sets with lower CDIs are more similar to the benchmark label set (Supplementary Fig.~S10).”*

*To take another example, in line 341 of page 13, we apply CDI to the CORTEX dataset, which has eight main types and 33 subtypes.*

*“CDI-BIC selected a 20-cluster label set (Fig. 4A CORTEX panel). These 20 clusters correspond to the partitions of eight main types in the benchmark label set…”*

*“CDI-AIC selected a label set with 36 clusters (Fig. 4A CORTEX (AIC) panel). Some of these 36 clusters were partitions of the benchmark subtypes…”*

*Although the cluster numbers of the identified label sets do not match the high-quality benchmark label sets, the CDI-identified label sets are similar to the high-quality benchmark label sets, measured by the external indices. See Figure 5B.*

*To sum up, a label set with the correct cluster number is not necessarily a high-quality label set and vice versa. In fact, the concept of “correct cluster numbers” is ill-defined. A cell type can always be further split into sub-clusters based on functional differences, developmental or activation stages, cell cycles, senescence level, etc. We developed CDI as an internal index to measure label set quality, and evaluation of the quality of a candidate label set is based on its similarity to the high-quality benchmark label set.*

*Second, we also agree with the reviewer that external indices are not always better than internal indices as their performance depends on the quality of the benchmark label set. If the benchmark label set is poor, the external indices will also select low-quality label sets. On line 29 of page 2, we mention that*

*“If a high-quality benchmark label set is available, external indices are more accurate than internal indices, which do not utilize the high-quality benchmark label set. However, external indices cannot be applied when the benchmark label set is unavailable. Moreover, external indices may perform unsatisfyingly when the benchmark label set is of low quality. These practical issues could occur during scRNA-seq clustering.”*

*On line 212 of page 8, we distinguish between the use of true label sets (for simulated data sets) or rigorously evaluated benchmark label sets (for moderate-scale experimental data sets) from the less reliable benchmark label sets (for the large-scale experimental data sets):*

*“All datasets have benchmark label sets. All simulated datasets use their true label sets as the benchmark label sets. For three moderate-scale datasets, T-CELL (2,989 cells), CORTEX (7,390 cells), and RETINA (26,830 cells), the benchmark label sets were obtained by the experimental and bioinformatics analysis process, including fluorescence-activated cell sorting (FACS), known feature gene checking, cell screening, and clustering (Methods: Experimental datasets). These benchmark label sets are of high quality. Although they are not the actual label sets, they reflect our best knowledge. For the two large-scale datasets, IPF (114,696 cells) and COVID (1,251,200 cells), annotating cells is very challenging. The large-scale cell population is often highly heterogeneous with hierarchical cell structures; thus, generating reliable clustering results is difficult. Also, traditional verification approaches like manually checking gene markers are too laborious for large-scale datasets. Thus, the benchmark label sets might not be as reliable.”*

*In our paper, the actual label sets of the simulated datasets and the benchmark label sets of T-CELL, CORTEX, and RETINA are of high quality. Thus, we use the external indices to evaluate the similarity between the candidate and benchmark label sets and treat them as gold standard measures. However, for IPF and COVID, we did not use the external indices as gold standards. We explained the reasons on line 371 of page 13. The benchmark label sets for IPF and COVID are mainly used to evaluate CDI’s computation time. See line 455 on page 17.*

*In response to the reviewer’s useful feedback, we have added a new paragraph explicitly explaining the objective and evaluation of CDI on line 534 of page 20,*

*“Second, CDI is not a clustering method; instead, it is an index to evaluate the quality of the candidate label sets: the lower the better. To demonstrate CDI is reliable, we used the external indices to evaluate the similarity between the candidate and the high-quality benchmark label sets and treated them as golden standards. CDI provides a complementary clustering evaluation analysis to the existing approach by focusing on general clustering quality rather than the correct cluster number. [50]”*

*Reference*

*50. Yu, L., Cao, Y., Yang, J.Y.H., Yang, P.: Benchmarking clustering algorithms on estimating the number of cell types from single-cell RNA-sequencing data. Genome Biol 23(1), 49 (2022). doi:10.1186/s13059-022-02622-0*

**Third round of review**

**Reviewer 1**

I have no further comment

**Reviewer 3**

The authors have addressed my comments. I have no further suggestions.
